# Integrin-guided development of defined supramolecular hydrogels for human kidney tubuloid culture

**DOI:** 10.1039/d6bm01010e

**Published:** 2026-11-03

**Authors:** Aurelius J. C. Roskothen-Shevchuk, Victor A. Veenbrink, Laura Rijns, Bram Bakker, Carla Pou Casellas, Jarno Drost, Marianne C. Verhaar, Patricia Y. W. Dankers, Gisela G. Slaats

**Affiliations:** a Department of Nephrology and Hypertension, University Medical Center Utrecht Utrecht The Netherlands G.G.G.Slaats@umcutrecht.nl; b Institute for Complex Molecular Systems, Eindhoven University of Technology Eindhoven The Netherlands; c Department of Biomedical Engineering, Eindhoven University of Technology Eindhoven The Netherlands; d Hubrecht Institute for Developmental Biology and Stem Cell Research, Royal Netherlands Academy of Arts and Sciences (KNAW) Utrecht The Netherlands; e Oncode Institute, Princess Máxima Center for Pediatric Oncology Utrecht The Netherlands; f Division Cell Biology, Metabolism & Cancer, Department Biomolecular Health Sciences, Faculty of Veterinary Medicine, Utrecht University Utrecht The Netherlands; g Department of Chemical Engineering and Chemistry, Eindhoven University of Technology Eindhoven The Netherlands; h SyMO-Chem Eindhoven The Netherlands

## Abstract

Tubuloids are epithelial organoids derived from tissue or urine that model tubular nephron segments of the kidney. Their use in preclinical research is limited by dependence on basement membrane extract (BME), a batch-variable animal-derived matrix that reduces reproducibility. Here, we evaluate defined supramolecular ureido-pyrimidinone (UPy) hydrogels as an alternative matrix for tubuloid culture. Re-analysis of a published tubuloid single-cell RNA-sequencing dataset guides selection of two ligands for UPy functionalization to support cell–matrix interactions: the αVβ3-binding cyclic RGD peptide (cRGDfK) and the laminin-derived IKVAV motif. Tubuloids are cultured in UPy gels functionalized with UPy-IKVAV, UPy-cRGDfK, or both, and compared with BME. UPy hydrogels and BME are comparably soft and predominantly elastic. Across experiments, tubuloid lines from six donors are evaluated by confocal imaging, gene expression analysis, and brightfield imaging. UPy-IKVAV hydrogels support cystic tubuloid formation, most closely resemble BME in morphology and lumen formation, and maintain a kidney marker profile comparable to BME after four days, whereas UPy-cRGDfK is associated with more irregular morphologies. Integrin β1 blockade disrupts lumen integrity in BME and UPy-IKVAV hydrogels, supporting a role for β1-mediated cell–matrix interactions in tubuloid organization. UPy-IKVAV hydrogels therefore support viable cystic tubuloids and show promise as a robust matrix for preclinical tubulopathy research.

## Abstract 2

Integrin-guided ligand selection identified IKVAV-functionalized supramolecular UPy hydrogels as a defined, animal-free matrix supporting cystic, basal-out human kidney tubuloids.

## Graphical abstract

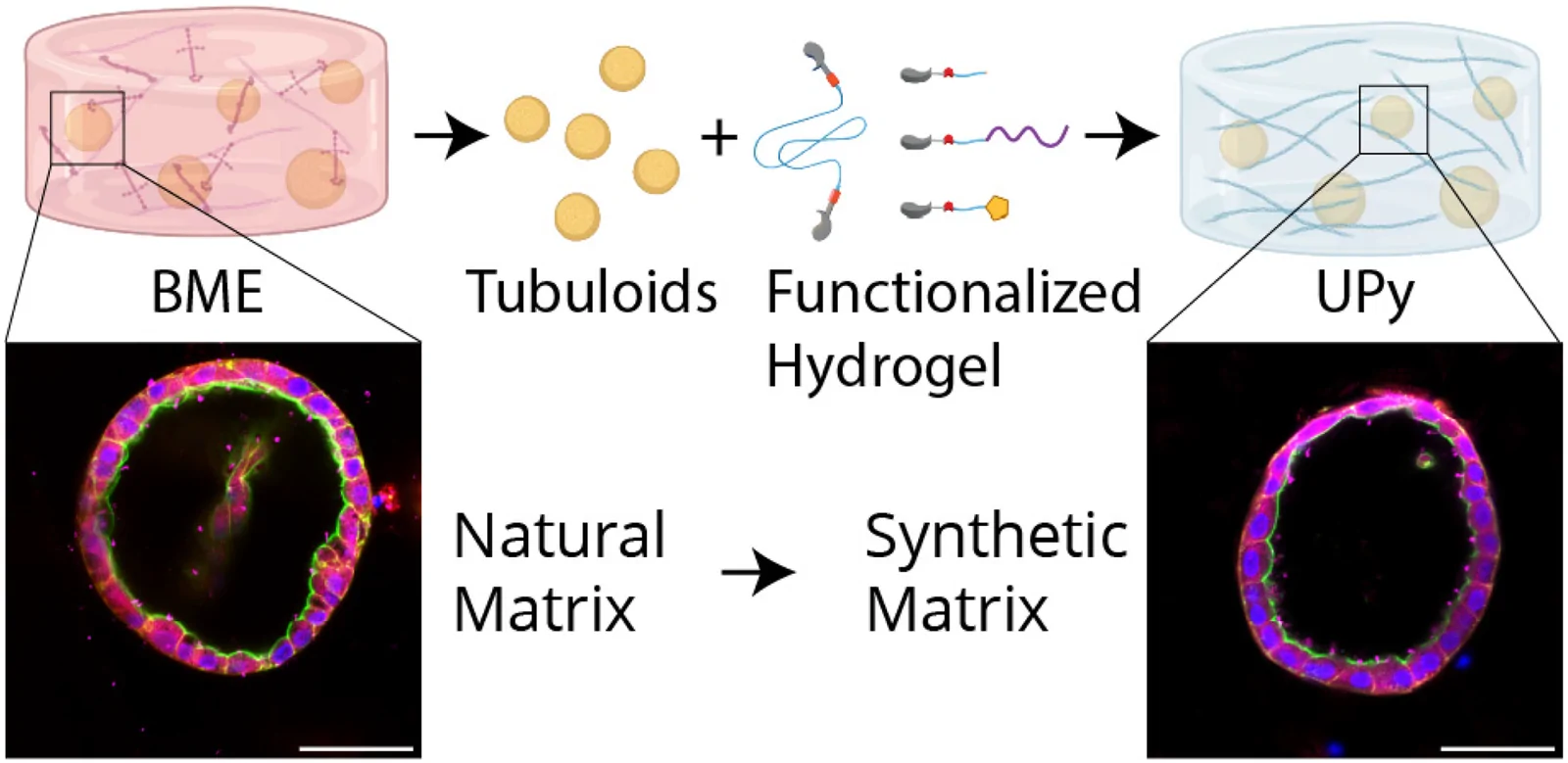

## 1. Introduction

Tubuloids are three-dimensional (3D) kidney epithelial organoids that represent the tubular segments of the nephron. They are characterized by a polarized epithelial monolayer which expresses classical tubular markers and displays primary cilia. Cultures can be derived from urine or kidney tissue from both healthy donors and patients, which enables patient-specific disease modeling and drug screening.^1,2^ Tubuloids are increasingly utilized to model human pathologies, hereditary diseases, malignancies and infections.^1,3–6^ Furthermore, tubuloids support nephrotoxicity assessments and microfluidic-based transport studies.^7,8^

Tubuloids are cultured in basement membrane extract (BME), also known as Cultrex™ or Matrigel™, a matrix derived from Engelbreth–Holm–Swarm mouse sarcoma tumors.^9–11^ BME is a poorly defined mixture of extracellular matrix (ECM) components and growth factors whose batch-to-batch variability can confound experimental outcomes.^12,13^ Key basement membrane components, including laminin-111, collagen IV, and fibronectin, support epithelial organoid growth through interactions with integrin heterodimers at the cell surface, thereby promoting the characteristic basal-out, apical-in polarity.^9,14,15^ Integrins are obligate α/β transmembrane heterodimers that mediate cell–ECM interactions and contribute to the establishment and maintenance of apical–basal polarity. Based on ligand specificity, they can be classified into RGD-binding, laminin-binding, collagen-binding, and leukocyte-specific receptors. In epithelial organoids, integrins localize predominantly to the basal, matrix-facing side.^1,15,16^

The animal-derived origin of BME, together with the limited ability to precisely control its physical and biochemical properties, has driven efforts to develop alternative matrices for epithelial organoid culture. These include fibrin-based matrices supplemented with laminin-111,^17^ invasin-functionalized polyisocyanide (PIC) gels,^18^ and synthetic hydrogel systems based on polyethylene glycol (PEG) maleimide or alginate.^19–21^

Recently, it was demonstrated that ureido-pyrimidinone (UPy)-based hydrogels, functionalized with the laminin-111-derived peptide IKVAV and supplemented with the TS2/16 integrin β1-stimulating antibody, support basal-out intestinal organoid growth. In the same study, blocking integrin β1 shifted intestinal organoids to an apical-out phenotype, confirming the essential role of integrin-mediated adhesion in controlling the epithelial polarity of intestinal organoids.^22^

UPy motifs form assemblies through a combination of supramolecular interactions, starting by dimerization through quadruple hydrogen bonding between monomers, followed by the stacking of dimers through a combination of π–π stacking, lateral hydrogen bonding between urea groups and hydrophobic interactions.^23^ Glycinamide functionalized UPy (UPy-G) has been shown to form fibers in aqueous environments several µm in length.^24^ When combined in a modular fashion with a crosslinker consisting of two UPy units and a PEG10K hydrophilic spacer (B-UPy), it can form a supramolecular hydrogel.^25^ This hydrogel can be made cell-adhesive through the modular incorporation of peptide-functionalized UPys such as UPy-cRGDfK and UPy-IKVAV (Fig. 1A).^22,25^

**Fig. 1:**
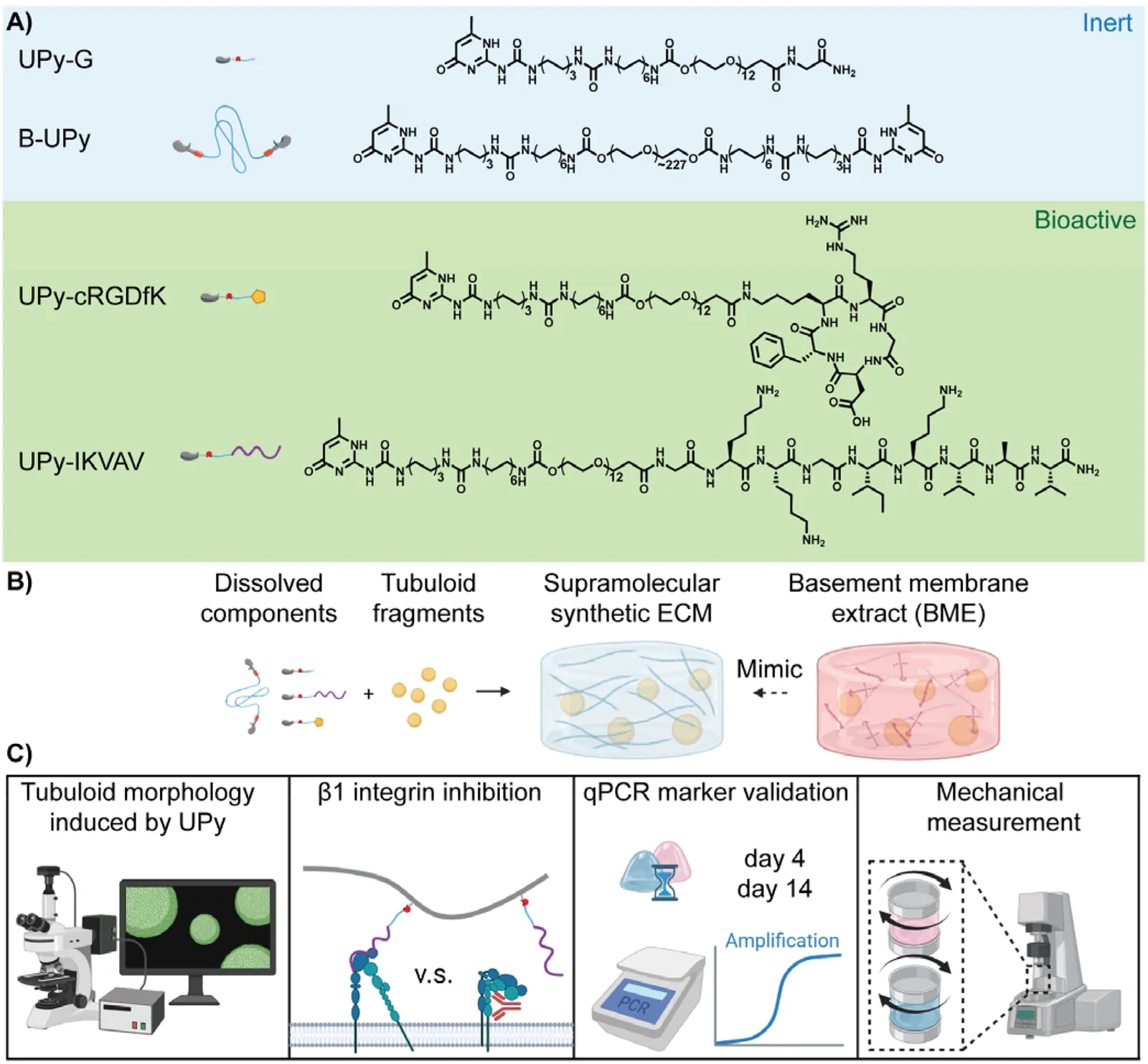
Overview of the approach used in this study to create ECM mimicking hydrogels. (A) Supramolecular components used to formulate the hydrogels, including peptide sequences that are attached to inert UPy units to mimic a bioactive response. (B) Schematic showing how dissolved monomers are mixed with tubuloid fragments. The mixture is then crosslinked to form a supramolecular ECM that encapsulates the tubuloid fragments (created with BioRender.com). (C) Overview of the approach used to develop BME-mimicking materials. UPy hydrogels were first screened for their ability to support cystic tubuloid morphology, after which the best-performing formulations were selected for further study. These were then evaluated for integrin β1-mediated interactions, kidney marker-gene expression, and mechanical properties in comparison with BME.

In this study, we evaluated whether supramolecular UPy hydrogels can support tubuloid cultures. Tubuloid fragments were resuspended in a mixture of culture medium and molecularly dissolved UPy components, which self-assemble into a stable hydrogel upon mixing (Fig. 1B). The capacity of UPy hydrogels to mimic the BME matrix was evaluated by investigating tubuloid polarity, morphology, and kidney marker mRNA expression levels, as well as the rheological properties of the respective gels (Fig. 1C).

## 2. Results and discussion

### 2.1. Tubuloid integrin profile indicates laminin and RGD affinity

Integrins localize to the basal, matrix-facing side of epithelial cells, where they mediate attachment to the surrounding matrix (Fig. 2A).^15,16^ To identify the integrin (ITG) subunits most relevant to tubuloid biology, we re-analyzed a published single-cell RNA-sequencing dataset of human kidney tubuloids in expansion medium.^4^ Among β subunit genes, *ITGB1* (integrin β1) showed the highest mean expression across the scRNA-seq dataset, followed by *ITGB6* (β6), *ITGB8* (β8), and *ITGB4* (β4). Among α subunit genes, *ITGA3* (integrin α3) showed the highest mean expression, followed by *ITGAE* (αE), *ITGA2* (α2), and *ITGAV* (αV) (Fig. 2B). Of the most highly expressed integrin subunits, α3 forms a unique heterodimer with β1, whereas β6 and β8 form unique heterodimers with αV.^16,26^

**Fig. 2:**
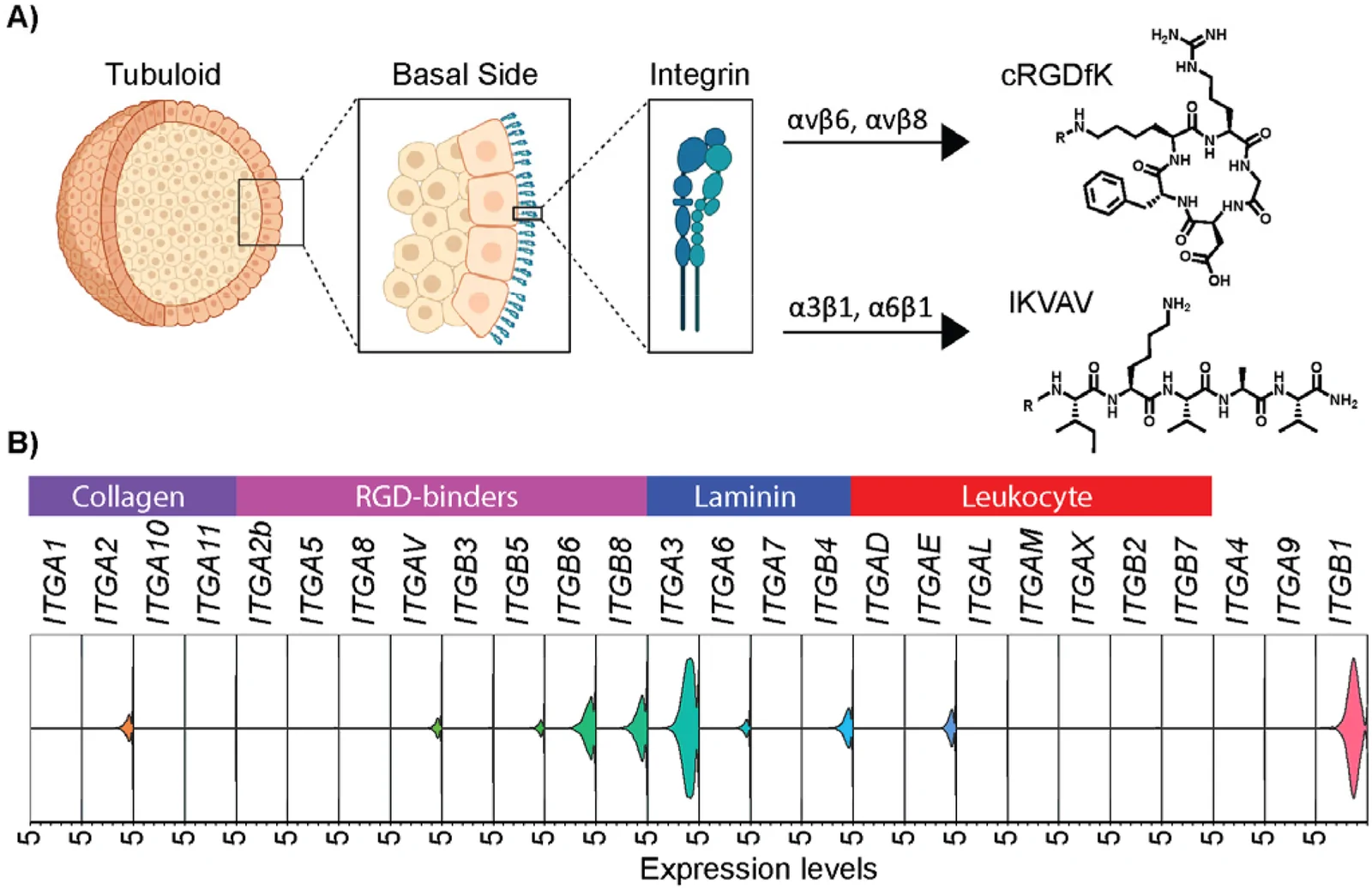
Integrin-guided selection of peptides for hydrogel functionalization. (A) Overview of integrin localization in tubuloids as well as integrin binding peptide sequences and their targets (created with BioRender.com). (B) Stacked violin plots showing transcript expression of integrin subunits based on re-analysis of a previously published tubuloid scRNA-seq dataset in expansion medium.^4^ Genes are grouped by ligand classes (collagen, RGD-binders, laminin, leukocyte) according to Pang *et al*.^16^

Based on these findings, two peptides were selected for hydrogel functionalization. The first chosen peptide was the cyclic RGD peptide cRGDfK, which predominantly targets αVβ3 integrin (IC_50_ = 2.25 nM), with weaker inhibition of αVβ6 (55 nM), α5β1 (141 nM), and αVβ5 (340 nM), and the lowest measured activity toward αVβ8 (5200 nM) and αIIbβ3 (>10 µM), as determined in a competitive integrin-binding assay.^27^ The second peptide was the laminin-111-derived IKVAV motif, which has been reported to engage α3β1 and α6β1,^28^ and to a lesser extent α2β1, although its exact binding affinity has not yet been quantified.^29^ Because the transcriptomic analysis revealed minimal expression of collagen-binding or leukocyte-associated integrins, no collagen-derived or leukocyte-specific peptides were included in the experimental design.

Since tubuloids represent the tubular segments of the kidney, their expression profile and our choice of ligand-derived peptide sequences align with integrin expression patterns observed *in vivo*. In human kidneys, integrin α6β1 is consistently expressed throughout the proximal tubule, distal tubule, and collecting duct, whereas α6β4 is only present in the collecting duct.^14^

### 2.2. Tubuloids display basal-out polarization in UPy conditions

Tubuloids were cultured in supramolecular hydrogels formed by co-assembly of monofunctional components (M-UPy : UPy-G and UPy-peptides) with a bifunctional crosslinker (B-UPy), using an 80 : 1 M : B molar ratio. This ratio was kept constant across all gel conditions. The total M-UPy concentration was chosen to be comparable to that of a 1.25% (w/v) reference gel consisting of UPy-G and B-UPy; as a result, peptide-containing gels had a slightly higher total solids content (up to 1.39% (w/v)). Because peptide-functionalized UPys substitute for UPy-G rather than being added to it, the total monofunctional UPy concentration was held constant at 9.38 mM across all formulations; the bioactive fraction within the fibers was therefore 10.66 mol% in the single-ligand gels and 21.32 mol% in the dual-ligand gel, at constant fiber and crosslink density.

Tubuloid morphology was quantified using morphology classification (Tables 2 and 3) and counting tubuloids with correct polarity. Here, recapitulation of BME was defined operationally as formation of tubuloids with (i) a cystic morphology, (ii) correct basal-out polarity, and (iii) a comparable distribution of round structures across size classes relative to BME. The peptide-functionalized UPys UPy-cRGDfK and UPy-IKVAV were tested individually (1 mM) and in combination (1 mM each; 2 mM total UPy-peptide). Three donor lines were included to capture biological variability. Confocal imaging was performed on day 4, with an additional brightfield endpoint on day 8 for a single donor to assess longer-term stability (Fig. S1).

In BME, UPy-IKVAV and UPy-cRGDfK & UPy-IKVAV conditions, tubuloids formed round structures with basal-out polarity, as indicated by apical localization of F-actin and primary cilia, and by basal localization of integrin β1 (Fig. 3A & Fig. S2). Quantitative classification confirmed that cultures in BME consisted primarily of round tubuloids (86.0 ± 6.9% by combined count, mean ± SD of three donors; Fig. 3B) and that most measured tubuloid area (79.5 ± 8.7%) was attributable to round tubuloids in the large-size class, consistent with tubuloid fragments being able to expand into larger structures in BME.

**Fig. 3:**
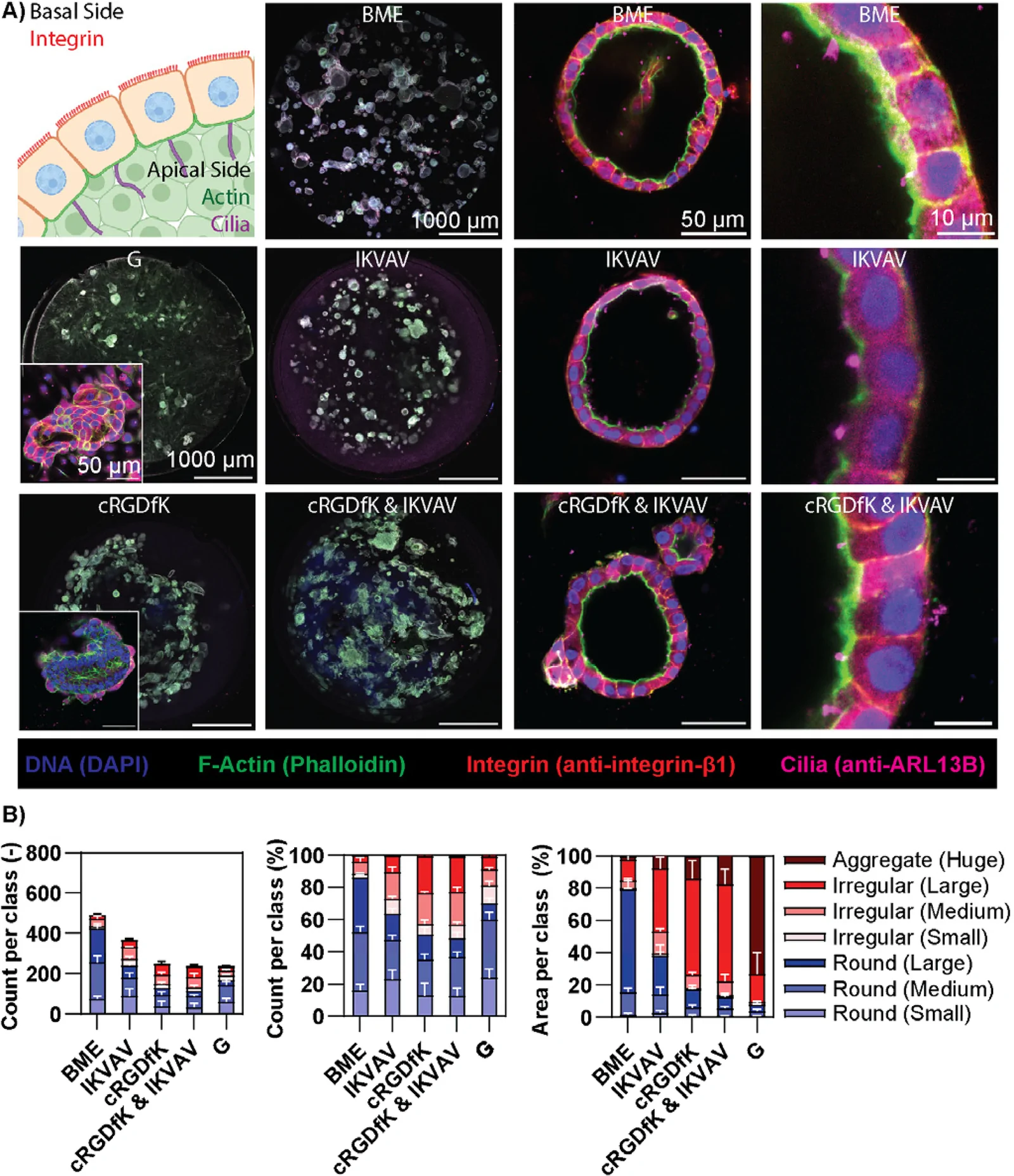
Overview of tubuloids cultured in supramolecular UPy hydrogels and BME. (A) Tubuloids were stained for nuclei (DAPI, blue), F-actin (phalloidin, green), integrin β1 (anti-integrin-β1, red), and cilia (anti-ARL13B, magenta). The first panel shows a schematic cross-section of a correctly polarized tubuloid (apical-in/basal-out), created with BioRender.com. For G and cRGDfK, representative full-well overviews are shown with higher-magnification insets. For BME, IKVAV, and cRGDfK & IKVAV, each condition is shown as a full-well overview, a single-tubuloid view, and a cross-section zoom to visualize apical–basal marker localization. Scale bars: 1000 µm (overview), 50 µm (single tubuloid/insets), and 10 µm (cross-section). UPy hydrogels were formulated at 80 : 1 M : B with UPy-G and 0–2 mM UPy-peptide (10.66 mol% each when present); solids were matched to a 1.25% (w/v) UPy-G reference gel. All images represent donor 1. (B) Distribution of count per class (absolute and %) and area per class for all outlines per image (mean ± SD across donors 1–3; 179–557 outlines per donor).

To determine whether cell-binding ligands were required, a UPy hydrogel lacking bioactive components (UPy-G only) was included as a negative control. In the absence of peptide ligands, tubuloids settled and spread along the plate surface, forming a 2D cell sheet rather than maintaining tubuloid morphology. Quantitatively, this shift was reflected by a large fraction of structures classified as aggregates (73.0 ± 28.6%; outlines with an area greater than ∼0.4 mm^2^). While several cystic organoid-like structures were still observed on top of the cell layer after four days, these collapsed by day 8 (Fig. S1). Notably, this 2D sheet-forming behavior was not observed in any of the bioactive hydrogels under the conditions tested.

Incorporation of cRGDfK led to large, irregular structures that lacked a cystic morphology and did not display correct basal-out polarity (Fig. 3A). Quantitatively, the irregular (large) class dominated total tubuloid area (59.5 ± 20.2%), and the fraction of round structures by count was reduced relative to BME (50.8 ± 12.0%) (Fig. 3B). By contrast, the laminin-111-derived UPy-IKVAV supported formation of a mixed population dominated by correctly polarized round structures (64.7 ± 10.0% by combined count; Fig. 3B). However, a substantial fraction of total tubuloid area remained attributable to large irregular and aggregate morphologies (47.0 ± 23.4% by combined area), indicating that while IKVAV presentation supported formation of correctly polarized, round tubuloids with a cystic morphology, it did not fully prevent the presence of large irregular and aggregate structures under these conditions (Fig. 3B).

To test whether multi-ligand presentation could improve this outcome, a multi-component gel containing both UPy-IKVAV and UPy-cRGDfK was evaluated (1 mM each; 2 mM total peptide-UPy). Dual-ligand presentation did not yield an improvement relative to single-ligand conditions based on the primary quantitative readouts: the round fraction by count was similar to UPy-cRGDfK alone (48.7 ± 4.1% round by count for UPy-cRGDfK & UPy-IKVAV *vs*. 50.8 ± 12.0% for UPy-cRGDfK), and total area remained dominated by the large irregular class to a comparable extent (60.0 ± 16.5% for UPy-cRGDfK & UPy-IKVAV *vs*. 59.5 ± 20.2% for UPy-cRGDfK) (Fig. 3B). Correctly polarized round tubuloids could still be observed in the dual-ligand gels, which was not the case for UPy-cRGDfK alone (Fig. 3A). Overall, the combined morphological data indicate that UPy-cRGDfK may partially offset the beneficial effects observed with UPy-IKVAV alone.

To provide a compact, donor-matched measure of overall morphology similarity to BME, the class distributions (by count% and by area%) were compared to the paired BME controls using the Jensen–Shannon distance (JSD) on the concatenated distribution vector (0 = identical; larger values = more dissimilar).^30^ By this metric, UPy-IKVAV was the closest UPy formulation to BME (0.379 ± 0.097, mean ± SD across donors), followed by UPy-cRGDfK (0.511 ± 0.101), UPy-cRGDfK & UPy-IKVAV (0.552 ± 0.083), and UPy-G (0.589 ± 0.113). This quantitative ranking aligned with the qualitative phenotypes, as IKVAV-containing gels were the only conditions that reliably produced a large fraction of tubuloids with a cystic morphology and correct polarization.

One possible explanation for the distinct phenotypic outcomes observed in the UPy-IKVAV and UPy-cRGDfK conditions is that the two ligands may preferentially engage different integrin–signaling modes, which in turn differentially regulate epithelial polarity and lumenogenesis.^31^ IKVAV is often described as a laminin-mimetic cue that can recruit β1-containing laminin receptors (notably α3β1 and α6β1), commonly associated with basement-membrane adhesion, stable cortical organization, and maintenance of epithelial architecture.^16,32^ By contrast, the enrichment of *ITGB6* and *ITGB8* in the reference single-cell dataset of BME-cultured tubuloids^4^ (Fig. 2) indicates that these cells have the transcriptional capacity to assemble αV-based integrins (αVβ6/αVβ8). On the assumption that this capacity is retained in our cultures, αV-based engagement offers a candidate explanation for the cRGDfK phenotype: αVβ6 and αVβ8 engagement is often linked to stronger focal-adhesion maturation and contractility (*e.g.*, through FAK/Src–RhoA/ROCK–actomyosin signaling), and to coupling of adhesion with TGF-β pathway activity, since both are implicated in local activation of latent TGF-β.^33,34^

Under this framework, cRGDfK presentation would be expected to bias cells toward a more αV-dominant, contractile or remodeling-prone state that is compatible with maintaining 3D attachment yet less permissive for stable apical–basal polarity and lumen maintenance, yielding irregular aggregates.^35,36^ Notably, both peptides were presented at millimolar (*i.e.*, supraphysiological) levels, which could amplify these effects by driving near-saturating receptor occupancy, enhanced integrin clustering/avidity, and competition or “integrin switching” between β1- and αV-mediated programs. Such density-dependent effects could also help explain why co-presentation did not improve morphology relative to IKVAV alone.^37^

Within the present dataset, ligand identity rather than total ligand content appears to be the dominant variable. The dual-ligand gel did not behave as an intermediate between the two single-ligand formulations, but reproduced the UPy-cRGDfK condition across all primary readouts, indicating that the phenotype followed the presence of cRGDfK rather than the size of the bioactive fraction. The opposite direction is not supported either: in the absence of any bioactive ligand, tubuloids failed to maintain their morphology altogether (73.0 ± 28.6% aggregates in UPy-G gels). Because ligand identity and effective ligand concentration co-vary between the single- and dual-ligand gels (10.66 *vs*. 21.32 mol%), a contribution of total ligand density cannot be formally excluded. Systematic variation of effective UPy-cRGDfK concentration from 0 to 44 mol% in the same UPy chemistry has, however, been reported to improve polarized cyst formation monotonically, albeit in a different epithelial cell system and stiffness regime, giving no indication that a higher bioactive fiber fraction is in itself detrimental.^31^

### 2.3. Integrin β1 inhibition disrupts lumen integrity in a formulation-dependent manner

Integrin β1 is a major mediator of epithelial cell–ECM interactions in the kidney.^14^ To assess whether the effects observed in the functionalized hydrogels depend on β1-mediated interactions, tubuloids were cultured in the presence of an integrin β1-blocking antibody (AIIB2, 2 µg mL^−1^; Fig. 4A). Under β1-inhibited conditions, tubuloids frequently displayed multi-lumen phenotypes and/or loss of a single continuous lumen in confocal cross-sections (Fig. 4B), consistent with disrupted cyst architecture. The representative images in Fig. 4B illustrate the two scoring categories used for the quantitative analysis, rather than their relative frequency or severity. Because lumen integrity was scored as a binary criterion, the apparent severity of individual structures in the representative images does not necessarily correspond to the magnitude of the change.

**Fig. 4:**
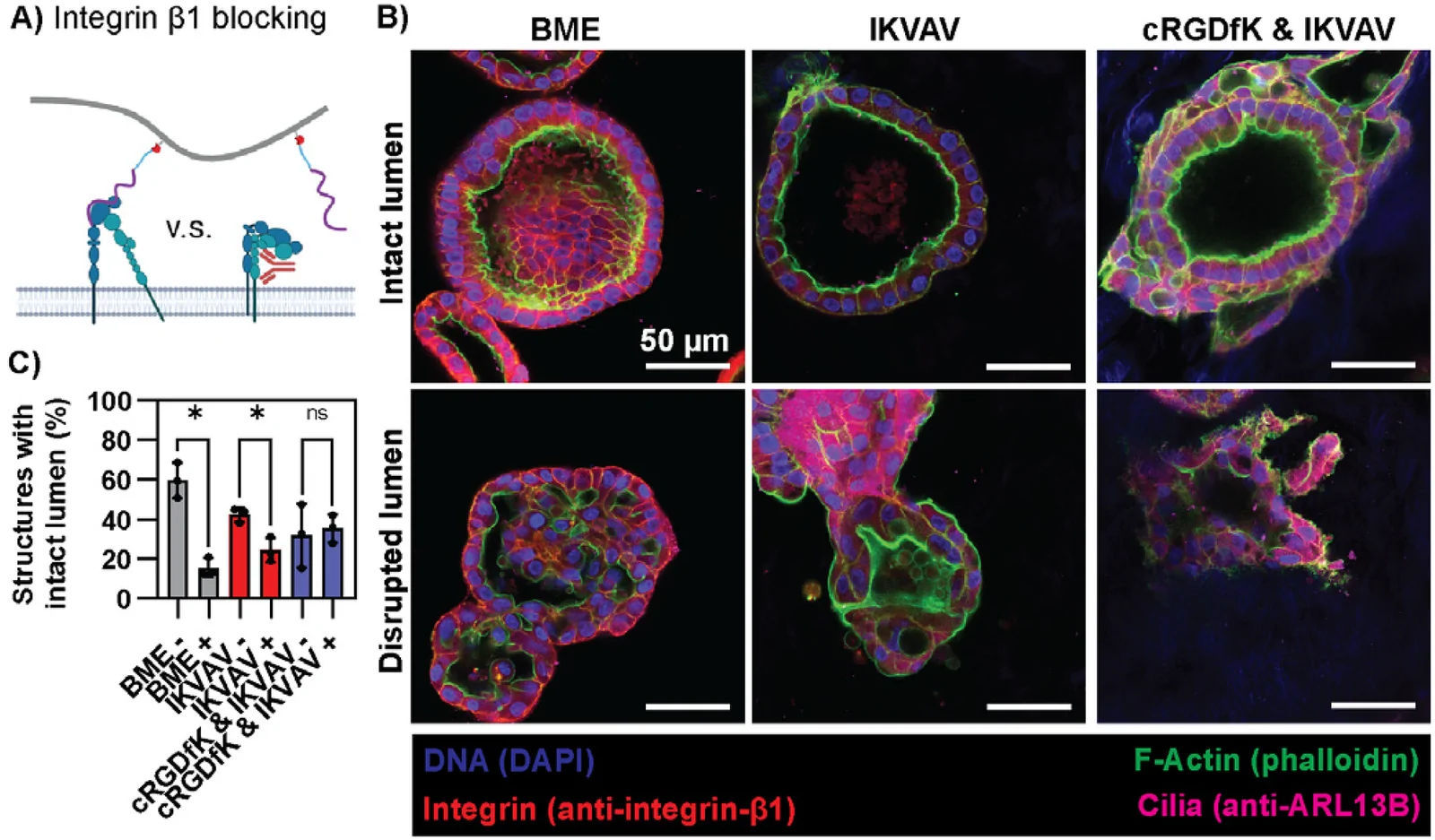
Tubuloids cultured under integrin β1-inhibiting conditions. (A) Schematic of integrin β1 inhibition by antibody binding (created with BioRender.com). (B) Representative tubuloids illustrating the two scoring categories used in (C): an intact lumen (top row) and a disrupted lumen (bottom row), shown for each hydrogel condition. Top-row images are from wells cultured without, and bottom-row images from wells cultured with, the integrin β1-blocking antibody AIIB2. Images illustrate phenotype and are not intended to convey the frequency with which each phenotype occurred; frequencies are quantified in (C). Samples were stained for nuclei (DAPI, blue), F-actin (phalloidin, green), integrin β1 (anti-integrin-β1, red) and cilia (anti-ARL13B, magenta); scale bar: 50 µm. UPy hydrogels were formulated at 80 : 1 M : B with UPy-G and 0–2 mM UPy-peptide (10.66 mol% each when present); solids were matched to a 1.25% (w/v) UPy-G reference gel. All images are from donor 1. (C) Percentage of all non-aggregate structures that were both round and had an intact lumen with correct polarization, cultured with (+) and without (−) integrin β1 inhibition. Intact lumen was scored as a binary criterion, defined as a single continuous lumen with correct apical–basal marker localization. Two replicate wells were scored per donor per condition and pooled within donor prior to analysis (23–177 structures per donor per condition; 1251 structures in total). Bars show mean and SD of donors 1–3; points represent individual donors. Ab− and Ab+ conditions were compared within each formulation by two-tailed paired *t*-test on donor-matched percentages, with Holm correction applied across the three formulations. Adjusted *p*-values: BME *p* = 0.012, UPy-IKVAV *p* = 0.029, UPy-cRGDfK & UPy-IKVAV *p* = 0.73. *p < 0.05, **p < 0.01, ns = not significant.

To quantify the effect of integrin β1 inhibition, the percentage of all non-aggregate structures that were both round and had an intact lumen with correct polarization was determined (Fig. 4C). In total 1251 structures were scored across three donors, with two replicate wells per donor per condition (23–177 structures per donor per condition); replicate wells were pooled within donor, and donor was treated as the biological replicate for the lumen-integrity analysis. Because baseline intact-lumen fractions differ substantially between matrices, the effect of β1 inhibition was assessed within each formulation using donor-paired measurements rather than by comparing absolute post-treatment fractions across formulations.

Integrin β1 inhibition reduced the intact-lumen fraction in both BME and UPy-IKVAV gels (BME: 59.7 ± 8.9% to 15.4 ± 4.8%; UPy-IKVAV: 42.4 ± 3.5% to 24.2 ± 6.2%; mean ± SD of three donors), with all three donors changing in the same direction in both formulations (paired *t*-test; Holm-adjusted *p* = 0.012 and *p* = 0.029, respectively, across the three formulation-specific comparisons). Relative to their own baselines, blockade reduced the intact-lumen fraction by 74.5 ± 4.4% in BME and 43.3 ± 10.7% in UPy-IKVAV.

In contrast, the UPy-cRGDfK & UPy-IKVAV condition showed no net reduction (31.7 ± 16.3% to 35.2 ± 7.2%; Holm-adjusted *p* = 0.73). This reflects discordance between donors rather than a uniform absence of response: two donors showed modest reductions of 15.2% and 11.8% relative to baseline, whereas the third increased from 15.0% (6/40 structures) to 36.2% (17/47), consistently across both replicate wells.

Despite these lumen abnormalities, the morphology distributions were largely preserved (Fig. S3). The morphology classifier (round: small/medium/large; irregular: small/medium/large; aggregate: huge) is based on 2D projected outlines from overview images and does not explicitly capture lumen number or lumen continuity, providing a straightforward explanation for why confocal lumen defects are not reflected as major shifts in class frequencies. Consistent with this, paired composite JSD between Ab− and Ab+ samples remained low across formulations (BME: 0.147 ± 0.050; IKVAV: 0.219 ± 0.123; cRGDfK & IKVAV: 0.209 ± 0.160; cRGDfK: 0.232 ± 0.099; G: 0.339 ± 0.153; mean ± SD across donors), indicating only modest changes in the overall shape/size-class distributions under β1 inhibition. In BME and UPy-IKVAV, apical–basal polarity markers remained appropriately localized in the examined structures, suggesting that the most apparent phenotype under β1 blockade was altered lumen architecture rather than an obvious loss of polarization.

Together, these results show that β1-mediated adhesion contributes to lumen integrity in tubuloids in a matrix-dependent manner, with consistent effects in BME and UPy-IKVAV. Because β1 pairs with twelve α subunits, blockade identifies a contribution of β1-dependent adhesion rather than implicating a specific heterodimer.

Two implications follow. First, tubuloids in UPy-IKVAV respond to β1 blockade in the same direction as those in BME, albeit with a smaller effect size. This supports a contribution of β1-dependent adhesion to lumen integrity in both matrices and provides functional support, beyond morphological similarity alone, for UPy-IKVAV as a defined BME alternative. Second, in contrast to the polarity reversal reported following β1 blockade in intestinal organoids,^22^ an analogous reversal of apical–basal marker localization was not apparent in the tubuloids examined here. Instead, the prominent phenotype associated with β1 blockade was disruption of lumen architecture.

### 2.4. UPy-IKVAV and UPy-cRGDfK & UPy-IKVAV functionalized-hydrogels support expression of key kidney markers comparable to that in BME-cultured tubuloids

To assess whether culture in the defined UPy hydrogels alters the kidney-associated transcriptional phenotype of tubuloids relative to BME, we quantified the expression of genes associated with tubular segment identity, epithelial state, progenitor/injury response, and proliferation in tubuloids cultured in BME or in UPy hydrogels functionalized with UPy-IKVAV or UPy-cRGDfK & UPy-IKVAV.

The genes selected to profile tubuloid gene expression and to represent distinct kidney tubular segments were based on published kidney biology literature and tubuloid expression data.^1,38,39^ On day 4, no significant differences in transcript levels were detected between tubuloids grown in BME and UPy-IKVAV or UPy-cRGDfK & UPy-IKVAV-functionalized hydrogels (Fig. 5A). These results indicate that both hydrogel conditions maintain a tubuloid marker profile comparable to BME at the 4-day time point. Notably, tubuloids cultured in the UPy-cRGDfK & UPy-IKVAV condition exhibited a trend toward increased expression of the proliferation marker *MKI67*.

**Fig. 5:**
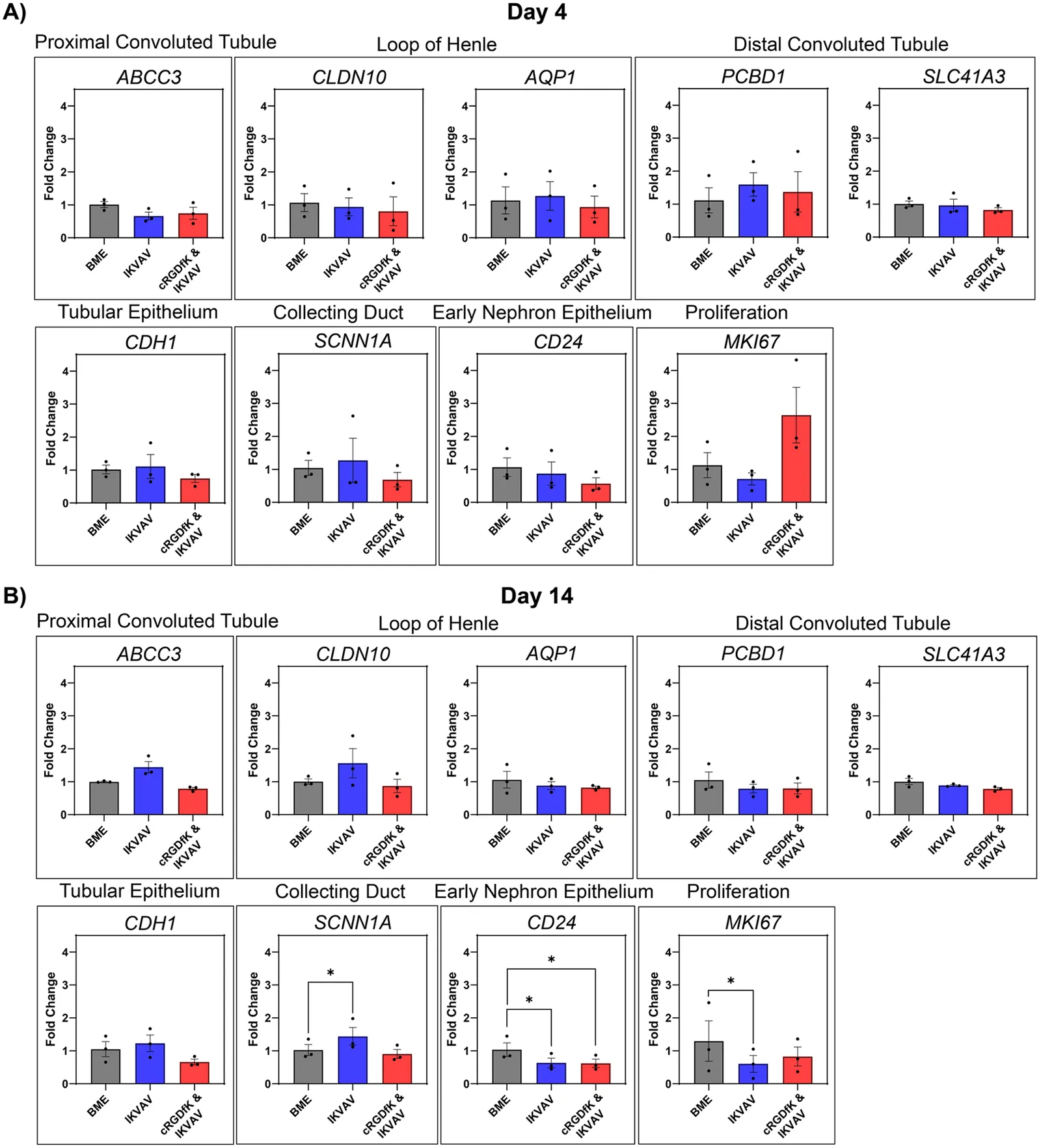
RT-qPCR analysis of tubuloid and nephron segment marker expression at day 4 and day 14. Expression of kidney markers of all tubular segments as well as epithelium, progenitor and proliferation markers assessed in tubuloids grown in UPy-biomaterials or BME. Kidney markers include: ATP-binding cassette sub-family C member 3 (*ABCC3*), Claudin-10 (*CLDN10*), Aquaporin-1 (*AQP1*), pterin-4α-carbinolamine dehydratase 1 (*PCBD1*), solute carrier family 41 member 3 (*SLC41A3*), cadherin-1 (*CDH1*), α-subunit of the epithelial sodium channel (*SCNN1A*), cluster of differentiation 24 (*CD24*) and marker of proliferation Antigen Kiel 67 (*MKI67*). All markers were analyzed at (A) day 4 and (B) day 14. Data are shown as mean ± SEM across donors (1–3) with technical duplicates averaged per donor, statistical significance: *p < 0.05, **p < 0.01. UPy hydrogels were formulated at 80 : 1 M : B with UPy-G and UPy-peptide (10.66 mol% each when present); solids were matched to a 1.25% (w/v) UPy-G reference gel.

To assess transcript level changes and the impact of the synthetic hydrogel environment over time, we analyzed the same marker panel on day 14 (Fig. 5B). Cystic tubuloid structures remained visible throughout the 14-day culture period (Fig. S4). Tubuloids cultured in UPy-IKVAV-functionalized hydrogels exhibited a significant upregulation of the collecting duct marker *SCNN1A*, which encodes a subunit of the ENaC channel, alongside a significant downregulation of *CD24* and *MKI67*, when compared to tubuloids cultured in BME. Similarly, tubuloids cultured in UPy-cRGDfK & UPy-IKVAV hydrogels also showed reduced *CD24* expression at day 14 (Fig. 5B).

Overall, these findings indicate that tubuloids cultured in UPy-IKVAV as well as UPy-IKVAV & UPy-cRGDfK hydrogels maintain a kidney marker expression profile comparable to BME on day 4. By day 14, tubuloids in both UPy conditions show reduced expression of *CD24*, a marker associated with kidney progenitor cells and linked to injury/repair responses, and implicated in tubuloid biology.^5,40^ However, the magnitude of these changes remains modest, with fold changes ranging from 0.44 to 0.92. This suggests that the overall tubuloid kidney marker expression is largely preserved in UPy hydrogels.

These findings are relevant for disease modeling, where preservation of tubulopathy-associated kidney marker expression is important for assessing protein localization in patient-derived tubuloids and to classify disease phenotypes. This strategy has recently been applied successfully in studies of tubulopathies and ciliopathies.^3,41^ Tubuloids cultured in UPy-IKVAV hydrogels maintained a marker expression profile comparable to BME until at least day 4 (Fig. 5), and showed clear cilia formation (Fig. 3 and S2). The retention of cystic morphology indicates that the gels continued to provide macroscopic three-dimensional support throughout the culture period. However, because UPy hydrogels are dynamic networks held together by reversible supramolecular interactions, monomer exchange, diffusion and cell-mediated remodeling may alter their composition, ligand presentation or mechanical properties over time. Long-term mass loss and rheological stability were not measured, and a contribution of such changes to the transcriptional differences observed at day 14 therefore cannot be determined. Overall, our results support UPy-IKVAV hydrogels as a promising platform for tubulopathy and ciliopathy research.

### 2.5. Bioactive UPy hydrogels have stiffness comparable to BME but show faster stress relaxation

The rheological properties of BME and bioactive UPy hydrogels functionalized with UPy-IKVAV or UPy-cRGDfK & UPy-IKVAV were compared to determine whether their different biological responses could be explained by bulk mechanics. All materials showed elastic-dominated behavior (*G*′ > *G*″; Fig. 6A and B and Fig. S5). At 1 rad s^−1^, both UPy hydrogels had storage moduli comparable to BME: *G*′ was 26.6 ± 11.1 Pa for UPy-IKVAV, 22.6 ± 5.0 Pa for UPy-cRGDfK & UPy-IKVAV, and 15.9 ± 0.3 Pa for BME (Fig. 6B). The UPy hydrogels showed a higher loss tangent than BME (tan(*δ*) = 0.206 ± 0.028 and 0.259 ± 0.022, respectively, *versus* 0.111 ± 0.008; Fig. 6C), indicating a larger viscous contribution under small-amplitude oscillatory shear.

**Fig. 6:**
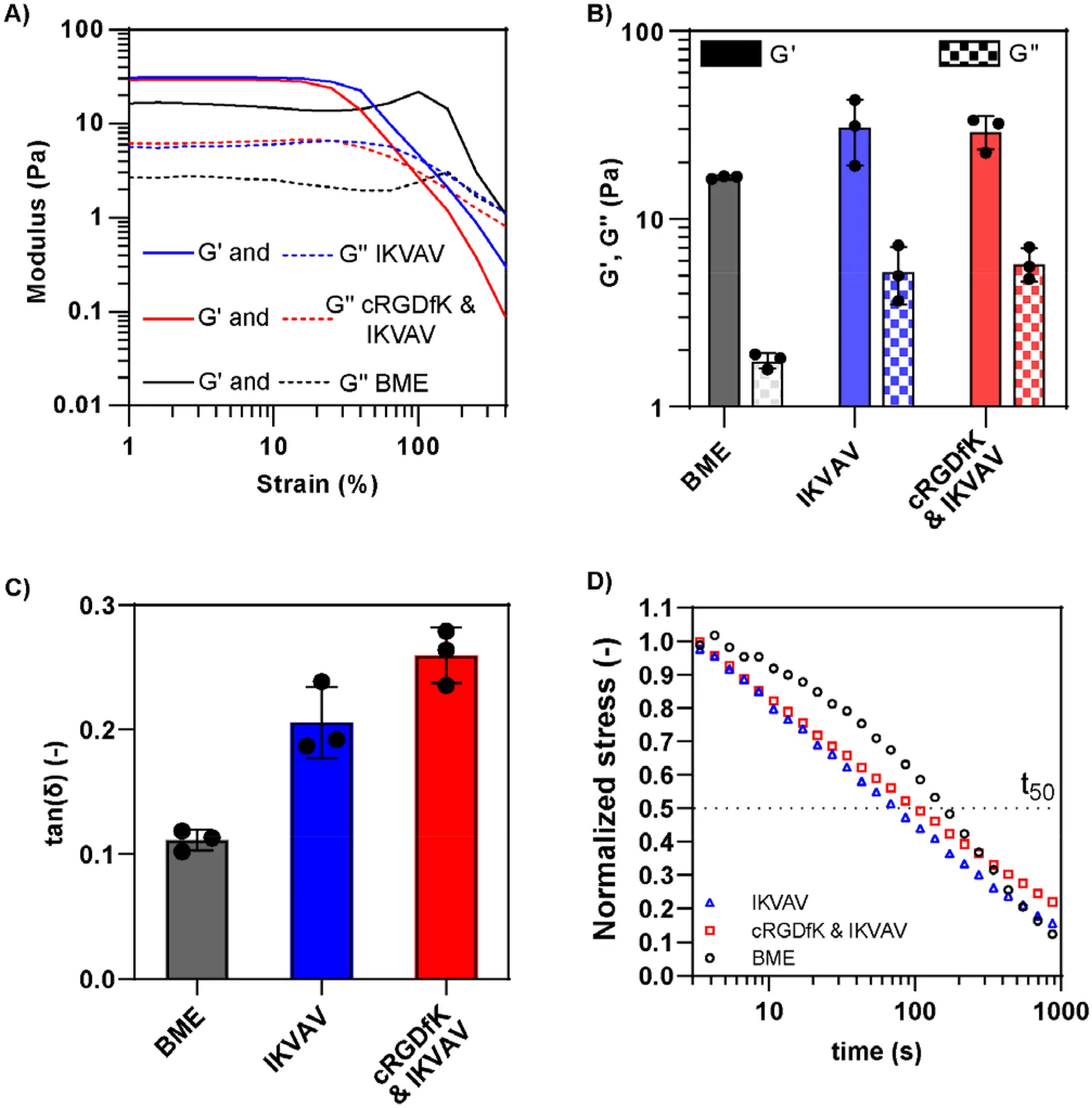
Rheological comparison of UPy biomaterials and BME. UPy hydrogels were prepared at a total concentration matched to a reference gel consisting of UPy-G and B-UPy at an 80 : 1 molar ratio and 1.25% (w/v). (A) Strain sweeps performed at 1 rad s^−1^. (B) Storage modulus (*G*′) and loss modulus (*G*″), and (C) loss tangent, tan(*δ*), obtained at 1 rad s^−1^ from frequency sweeps performed at 1% strain. (D) Stress-relaxation curves following application of 7.5% strain. Data acquired before 3 s were excluded because of instrument settling, and each trace was normalized to the stress measured at 3.0065 s. Curves are displayed as the mean of all data points falling within each of 30 logarithmically spaced time bins; binning was used for visualization only. *t*_50_ was defined as the first acquired time point at which normalized stress fell below 0.5 and remained below 0.5 for the following three acquired points. Lines in (A) and (D) represent the mean of three separately prepared gels. Bars in (B) and (C) show the mean and SD of three separately prepared gels.

The time required for the stress to decrease to half of its value was 276.8 ± 111.9 s for BME, 74.1 ± 16.1 s for UPy-IKVAV, and 82.7 ± 36.7 s for UPy-cRGDfK & UPy-IKVAV (Fig. 6D). Thus, although the materials were similar in bulk stiffness, both UPy hydrogels showed faster stress relaxation over the measured post-transient time window. Because the peak stress was not resolved before acquisition began, these values describe relaxation relative to the stress present at 3 s and should not be interpreted as peak-referenced relaxation half-times.

To assess the influence of bulk stiffness, additional UPy hydrogels with increased B-UPy content were prepared at B-UPy : M-UPy ratios of 1 : 40 and 1 : 20 (Fig. S6). Increasing the total polymer content through B-UPy increased *G*′ from 26.6 ± 11.1 to 71.7 ± 29.5 and 213.3 ± 175.1 Pa for UPy-IKVAV, and from 22.6 ± 5.0 to 45.6 ± 32.5 and 91.6 ± 61.5 Pa for UPy-cRGDfK & UPy-IKVAV at 1 : 80, 1 : 40, and 1 : 20, respectively. tan(*δ*) remained within a narrow range (0.188–0.259) across the UPy formulations.

Relaxation became slower with increasing B-UPy content. Post-transient *t*_50_ increased from 74.1 ± 16.1 to 109.5 ± 30.9 and 177.9 ± 25.7 s for UPy-IKVAV, and from 82.7 ± 36.7 to 138.9 ± 71.1 and 179.1 ± 90.5 s for the dual-ligand gels at 1 : 80, 1 : 40, and 1 : 20, respectively. Tubuloids from donors 4 and 5 formed cystic structures with apical-in polarity under all conditions, with no apparent stiffness-dependent differences. Therefore, within the investigated UPy stiffness and post-transient relaxation range, the observed phenotypic differences are unlikely to be explained solely by these bulk mechanical properties.

While the formulations showed comparable bulk viscoelastic properties, differences in nanoscale organization may also influence cell behavior. In aqueous environments, UPy-G assembles into micrometer-long fibers that are connected by B-UPy into a dynamic network.^24,25^ Fiber dimensions, bundling and mesh organization influence the spacing and accessibility of conjugated ligands and may thereby modulate integrin clustering, cell-generated forces, epithelial polarization, molecular transport and matrix remodeling. The hydrated architecture of the peptide-functionalized formulations was not resolved here, so peptide-dependent effects on co-assembly may contribute to the observed morphological differences. However, because UPy-G represented 78–89 mol% of the monofunctional components and the total monofunctional UPy concentration and M-UPy:B-UPy ratio were kept constant, we expect the overall fibrillar architecture to be determined primarily by the UPy-G/B-UPy scaffold.

### 2.6. Tubuloids in UPy-IKVAV display cystic morphology over six donor lines

Across the experiments described above, six different tubuloid donor lines were investigated. We observed differences between tubuloid donors, but all donor lines in UPy-IKVAV-functionalized hydrogels were able to form tubuloids (Fig. 7).

**Fig. 7:**
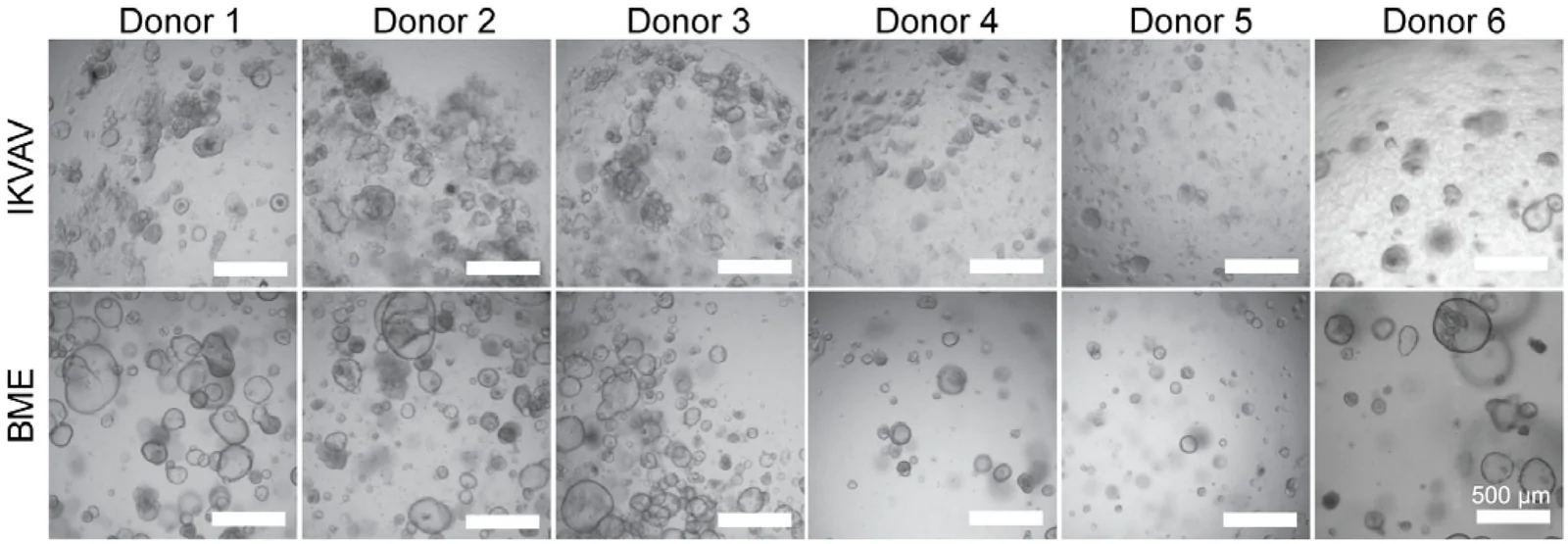
Day 4 brightfield comparison of tubuloids cultured in IKVAV hydrogels and BME across six donors. UPy-IKVAV supports cystic tubuloid morphology consistently across donors. Donor-to-donor variability in baseline morphology is illustrated by the corresponding BME cultures from the same donors. UPy-IKVAV-functionalized hydrogels were formulated at 80 : 1 M : B with 10.66 mol% UPy-IKVAV (1 mM) and 89.34 mol% UPy-G, and total solids were matched to a 1.25% (w/v) UPy-G reference gel. Scale bar: 500 µm.

In summary, these findings demonstrate that tubuloids from multiple donors can be robustly cultured in UPy-IKVAV hydrogels while preserving essential epithelial morphology, with inherent donor-specific variability. These features are readily captured using brightfield microscopy, enabling brightfield-based functional assays such as forskolin-induced swelling,^42^ as well as for disease modeling applications where gross morphological changes in tubuloids are informative.

## 3. Conclusions

Supramolecular UPy hydrogel outcomes depend on ligand identity. Among the tested formulations, UPy-IKVAV was the most robust condition, consistently generating cystic, basal-out tubuloids that closely recapitulated BME in both morphology distribution and marker gene expression. The apical localization of cilia further supports correct epithelial organization and highlights the potential of this system for ciliopathy research. In contrast, hydrogels containing UPy-cRGDfK produced a higher proportion of irregular structures with poor lumen formation. Dual-ligand presentation of UPy-cRGDfK & UPy-IKVAV did not improve morphology, although gene expression profiles remained similar to BME-cultured tubuloids, indicating that co-presentation shifts the system away from the desired phenotype rather than providing an additive benefit.

Blocking integrin β1 reduced the proportion of tubuloids retaining an intact lumen with basal-out polarity in BME and UPy-IKVAV hydrogels, indicating that β1-dependent adhesion contributes to lumen maintenance in both matrices. The UPy gels showed higher loss tangents and faster post-transient stress relaxation than BME, while remaining in a comparable soft, elastic-dominated regime. This supports the interpretation that the key phenotypic differences observed across conditions are more plausibly linked to biochemical cue presentation and matrix composition than to large differences in linear-regime stiffness. Overall, UPy-IKVAV-functionalized hydrogels are the best-performing defined formulation.

Our findings establish UPy hydrogels as a defined, modular, animal-free alternative to BME, and position them as a practical reference platform for tubuloid culture. By constraining the matrix design space to a limited set of well-defined, tunable parameters, UPy hydrogels enable systematic perturbation and a more direct link between compositional inputs and phenotypic outputs, even if they do not fully recapitulate every feature of BME. In this context, the present work not only demonstrates that tubuloids can be cultured in a fully defined hydrogel, but also establishes a modular culture platform for preclinical research where changes in matrix composition or culture conditions can be linked directly to the experimental intervention, rather than being confounded by batch variability or undefined components.

## 4. Experimental section

### 4.1. Integrin transcript expression

We re-analyzed a previously published single-cell RNA-sequencing dataset of tubuloids that was provided as a processed Seurat object (.RDS). The object contained normalized and integrated gene expression assays, dimensionality-reduction embeddings, and clustering assignments as generated in the original publication.^4^ No additional filtering, normalization, or batch-correction procedures were applied beyond those already present in the provided object. The stacked violin plot of integrin gene expression presented in this study was generated using the Seurat package (v5.3.0) in R (v4.5.1).

### 4.2. Tubuloid culture

Tubuloid cultures were generated from healthy kidney tissue obtained from donors following the protocol described by Schutgens *et al.*^1^ Tubuloid lines were used between passages 4 and 13 for all experiments.

For encapsulation into synthetic hydrogels, the basement membrane extract (BME) surrounding the tubuloids was first disrupted, after which the cultures were mechanically sheared to obtain fragments of uniform size. The sheared tubuloids were then washed twice with cold Advanced DMEM/F12 (Gibco) to remove residual BME. The resulting fragments were resuspended to yield a cell suspension comprising one-third of the final hydrogel volume for both control and experimental conditions, corresponding to a 1 : 3 passage ratio. Depending on the downstream assay, tubuloids were encapsulated in different hydrogel formats. For brightfield microscopy and RT-qPCR experiments, tubuloids were cultured in 60 µL hydrogels in U-bottom CELLSTAR® TC 96-well plates (Greiner Bio-One). For confocal microscopy experiments, tubuloids were cultured in thin hydrogels (10 µL) in ibidi µ-Plate 96 Well 3D plates. After combining the cell suspension with the hydrogel components, the constructs were incubated at 37 °C for 2 hours to allow complete gel polymerization. Medium changes were performed every other day.

Integrin inhibition was performed using an anti-integrin β1, AIIB2 (CD29) blocking antibody (Millipore, MABT409), applied at a final concentration of 2 µg mL^−1^. The antibody was added both to the hydrogel mixture during encapsulation and to the culture medium throughout the experiment.

### 4.3. Ureido-pyrimidinone gels

UPy-G, UPy-cRGDfK and bifunctional UPy-PEG10K-UPy (B-UPy) were obtained from SyMO-Chem BV (Eindhoven, The Netherlands) and synthesized as reported previously.^25^ UPy-IKVAV was synthesized in-house; its synthesis and characterization are described in ref. 22, and the same batch was used throughout this study. All compounds were used without further purification. Individual UPy compounds were weighed separately in aluminum weigh boats using a microbalance and transferred to clean glass vials. All compounds except UPy-IKVAV were dissolved in basic ultrapure water (adjusted to pH 12 with NaOH) under stirring at 60 °C for 30 minutes (UPy-G, UPy-cRGDfK 6× the concentration of the final gel, UPy-PEG10K-UPy (B-UPy): 18× the final concentration of the gel). UPy-IKVAV was dissolved in acidic ultrapure water (adjusted to pH 2 with HCl) under stirring at 60 °C for 30 minutes (6× the concentration of the final gel). Basic solutions of UPy-G and UPy-G + UPy-cRGDfK were neutralized by mixing with an equivalent volume of acidic solution with or without UPy-IKVAV, followed by an addition of an equivalent volume of 3× PBS (pH 7.4), resulting in a 2× concentrated solution of monofunctional UPy compounds. B-UPy solution was neutralized using the same steps resulting in a 6× concentrated solution. All neutralized solutions were subjected to sonication in a water bath for 5 minutes. Following sonication, the solutions were sterilized in open vials by UV exposure in a laminar flow cabinet for 10 minutes. Gels were formulated by mixing monofunctional UPy-solution with bi-functional UPy-solution and sheared tubuloids in culture medium in a volumetric ratio of 3 : 1 : 2, resulting in hydrogels with their final composition (Table 1). After combining the cell suspension with the hydrogel components, the constructs were incubated at 37 °C for 2 hours to allow complete gel polymerization.

**Table 1:** UPy-hydrogel compositions per gel type, with M-UPy : B-UPy molar ratio and total solids content (w/v%) in parentheses

Gel-type (M : B, w/v%)		UPy-G	UPy-cRGDfK	UPy-IKVAV	B-UPy
G (80 : 1, 1.25)	Ratio	80	0	0	1
	Conc. (mM)	9.38	0	0	0.12
	w/v%	1.12	0	0	0.13
IKVAV (80 : 1, 1.33)	Ratio	71.5	0	8.5	1
	Conc. (mM)	8.38	0	1	0.12
	w/v%	1	0	0.2	0.13
cRGDfK (80 : 1, 1.30)	Ratio	71.5	8.5	0	1
	Conc. (mM)	8.38	1	0	0.12
	w/v%	1	0.17	0	0.13
cRGDfK&IKVAV (80 : 1, 1.39)	Ratio	62.9	8.5	8.5	1
	Conc. (mM)	7.38	1	1	0.12
	w/v%	0.88	0.17	0.2	0.13
IKVAV (40 : 1, 1.46)	Ratio	71.5	0	8.5	2
	Conc. (mM)	8.38	0	1	0.23
	w/v%	1	0	0.2	0.26
cRGDfK&IKVAV (40 : 1, 1.51)	Ratio	62.9	8.5	8.5	2
	Conc. (mM)	7.38	1	1	0.23
	w/v%	0.88	0.17	0.2	0.26
IKVAV (20 : 1, 1.72)	Ratio	71.5	0	8.5	4
	Conc. (mM)	8.38	0	1	0.47
	w/v%	1	0	0.2	0.52
cRGDfK&IKVAV (20 : 1, 1.77)	Ratio	62.9	8.5	8.5	4
	Conc. (mM)	7.38	1	1	0.47
	w/v%	0.88	0.17	0.2	0.52

Gels for cell culture experiments were prepared directly in the appropriate well plate. For brightfield microscopy and qPCR experiments, 60 µL gels were prepared in U-bottom CELLSTAR® TC 96-well plates (Greiner Bio-One). For confocal microscopy experiments, 10 µL gels (approximately 800 µm thickness) were prepared in the inner well of a 3D µ-Plate (ibidi). For rheology measurements, 100 µL gels were prepared in parafilm indentations to facilitate transfer of the gels to the rheometer while minimizing mechanical damage. Gels were stored overnight at 37 °C, 5% CO_2_ in a humidified environment prior to measurement.

### 4.4. Immunofluorescence staining

Tubuloids were cultured and fixed directly in µ-Plate 96-Well 3D plates (ibidi) using 4% paraformaldehyde (PFA) for 45 min at room temperature (RT). Blocking and permeabilization were performed for 60 min at RT using 0.5% BSA and 0.5% Triton X-100 in PBS. Wash steps were carried out with 0.5% BSA and 0.1% Tween-20 in PBS. Primary antibodies were diluted in the same buffer and incubated overnight at 4 °C at the following concentrations: anti-ARL13B (Proteintech, 17711-1-AP; 1 : 400) and anti-CD29/integrin β1 (Thermo Fisher, clone TS2/16, 14-0299-82; 1 : 100). Secondary antibodies, consisting of goat anti-rabbit IgG Alexa Fluor™ 568 (Thermo Fisher, A-11011; 1 : 500) and goat anti-mouse IgG Alexa Fluor™ 680 (Thermo Fisher, A-21057; 1 : 500), were applied overnight at 4 °C. Phalloidin Alexa Fluor™ 488 (Thermo Fisher, A12379) and DAPI were added according to the manufacturers’ instructions and incubated for 45 min at RT. After staining, tubuloids were mounted in Mowiol. Imaging was performed using a Nikon Ti2 confocal microscope (4×/0.2 dry objective for overview images or 40×/1.15 water immersion with Nikon Spatial Array Confocal detector for single tubuloid images). Acquisition settings were held constant across conditions within each magnification. Because the hydrogel formulations differ in optical transparency and tubuloid size varies between structures, fluorescence intensity is not comparable between conditions; images are therefore interpreted for marker localization only.

### 4.5. Quantitative morphological measurements

All morphological measurements were performed on maximum-intensity projections (MIPs) of three z-slices (spaced 5 µm apart) in the F-actin (phalloidin) channel, acquired as 4×/0.2 overview images. For each condition, all visible structures were manually outlined in the overview images (two images per biological replicate; three biological replicates per condition). The resulting 2D outlines were then classified into seven morphology classes using shape descriptors computed from the projected outlines (circularity, aspect ratio (AR), roundness, and solidity) together with the outline area (px^2^).

Classification was performed in a sequential manner. First, very large structures were separated as aggregates: any outline with an area strictly greater than 20 000 px^2^ was assigned to the aggregate (huge) class. All remaining (non-aggregate) structures were then assigned to either a round or irregular shape group using an unsupervised two-component Gaussian mixture model (GMM) fit to standardized values of circularity, AR, roundness, and solidity. The component labelled “round” was selected by an objective rule that favors round outlines: the component maximizing median(circularity) + median(roundness) + median(solidity) − median(|AR − 1|) was labelled round, and the other component was labelled irregular. Using this procedure, 6174 non-aggregate structures were labelled round and 3237 were labelled irregular across the analyzed dataset. Finally, within each shape group (round and irregular), structures were partitioned into small/medium/large size categories using *k*-means clustering (*k* = 3) on log10(area). This yields two area boundaries per shape group, which can be interpreted as follows: small structures have area ≤ boundary 1, medium structures have boundary 1 < area ≤ boundary 2, and large structures have area > boundary 2. Combining the shape group and size bin yielded the final set of seven classes: round (small/medium/large), irregular (small/medium/large), and aggregate (huge). The summary statistics of the two GMM components and the resulting area cutoffs used for classification are reported in Tables 2 and 3.

**Table 2:** Median shape classifiers

Shape group	Circularity	AR	Roundness	Solidity
Irregular	0.732	1.668	0.600	0.921
Round	0.949	1.202	0.832	0.989

**Table 3:** Size classifier boundaries

Classification step	Shape group	Boundary 1 (px^2^|µm^2^)		Boundary 2 (px^2^|µm^2^)	
Aggregate (huge)	All	>20 000	>373 248	—	—
Size bins	Round	114.06	2129	435.34	8124
Size bins	Irregular	153.75	2869	981.88	18 324

### 4.6. Statistical analysis of lumen integrity

Intact-lumen fractions were scored per well and pooled within donor prior to analysis; the donor was treated as the unit of replication (*n* = 3). Within each formulation, Ab− and Ab+ conditions were compared using a two-tailed paired *t*-test on donor-matched percentages, on the percentage-point scale on which the outcome is reported throughout. Holm correction was applied across the three formulations.

### 4.7. Quantitative-PCR and expression analysis

For RT-qPCR experiments, tubuloids were cultured in 60 µL hydrogels in U-bottom CELLSTAR® TC 96-well plates (Greiner Bio-One). For each donor and condition, two to four technical culture replicates were pooled, resulting in one sample per donor (three donors in total). RNA extraction and cDNA synthesis were performed using the ISOLATE II RNA Mini Kit (Bioline) and the SensiFAST cDNA Synthesis Kit (Meridian Bioscience), respectively, following the manufacturers’ instructions.

Quantification of gene expression was carried out using the FastStart Universal SYBR Green Master (Rox) (Roche) on a BIO-RAD CFX384 detection system. Previously validated primer sets were used for all target and reference genes (Table 4).

**Table 4:** Primer sequences for gene expression measurements using RT-qPCR

Target gene	Forward sequence 5′ → 3′	Reverse sequence 5′ → 3′
*ACTB*	CATTCCAAATATGAGATGCGTTGT	TGTGGACTTGGGAGAGGACT
*ABCC3*	CACCAACTCAGTCAAACGTGC	GCAAGACCATGAAAGCGACTC
*CLDN10*	CCTGGGCTTCTTTGGTTCCA	TCATTGAGCACAGCCCTGAC
*AQP1*	CTGGGCATCGAGATCATCGG	ATCCCACAGCCAGTGTAGTCA
*PCBD1*	GGGTGGAATGAGCTGGAAGG	AGCGTGATGTGGACCTTGTT
*SLC41A3*	AGCCGAATCTCAACCTACCTG	CATGGAATTGATTTCTGACGTGC
*CDH1*	CGAGAGCTACACGTTCACGG	GGGTGTCGAGGGAAAAATAGG
*SCNN1A*	GCAGACGCTCTTTGACCTGTA	AGTCCGATTTGTTCTGGTTGC
*CD24*	CTCCTACCCACGCAGATTTATTC	AGAGTGAGACCACGAAGAGAC
*MKI67*	ACGCCTGGTTACTATCAAAAGG	CAGACCCATTTACTTGTGTTGGA

All downstream calculations were performed in R (version 4.5.1). Samples with a raw Cq standard deviation > 1 or a raw Cq > 38 were excluded from further analysis. For each gene, technical qPCR replicates were averaged to obtain a single Cq value per sample. ΔCt values were calculated by subtracting the Cq of the housekeeping gene (β-actin) from the Cq of the corresponding target gene. To normalize across donors, ΔCt values were further centered by referencing them to the pooled mean ΔCt of the control condition (BME), thereby retaining inter-donor variability, including within controls. Fold change values were computed using the 2^(−ΔΔCt)^ method. The normalized dataset was subsequently exported in a format suitable for statistical analysis and visualization in GraphPad Prism (version 10.6.1).

### 4.8. Statistical analysis of qPCR data

Statistical analysis and plotting were performed using GraphPad Prism version 10.6.1. ΔCt values were analyzed by one-way repeated-measures ANOVA, treating donors (*N* = 3) as matched subjects. The Geisser–Greenhouse correction was applied to account for potential unequal variances between conditions. When the overall ANOVA indicated significant differences (*p* < 0.05), Dunnett's multiple comparisons test was used to compare each condition with the control. Multiplicity-adjusted *p* values are reported, with significance defined as *p* < 0.05.

### 4.9. Rheology

Rheological measurements were performed on a Discovery Hybrid Rheometer (HR-20, TA Instruments) using an 8 mm stainless-steel parallel-plate geometry (800 µm gap) at 37 °C. Samples were protected from evaporation with low-viscosity silicone oil.

Hydrogel stability was assessed by a time sweep at 1% strain and 1 rad s^−1^. Frequency sweeps were performed from 100 to 0.1 rad s^−1^ at 1% strain. Stress relaxation was measured by applying 7.5% strain with a rise time of 0.05 s and monitoring the stress for 981 s. Strain sweeps were performed from 1 to 1000% strain at 1 rad s^−1^.

*G*′, *G*″, and tan(*δ*) were obtained at 1 rad s^−1^ from the frequency sweep. Stress-relaxation data acquired before 3 s were excluded because of an instrument-settling transient. Each trace was normalized to its value at the acquired time point closest to 3 s (3.0065 s). *t*_50_ was defined as the first acquired time point at which the normalized stress fell below 0.5 and remained below 0.5 for the following three acquired points. Values are reported as mean ± SD of three separately prepared gels.

### 4.10. Ethics statement

This study was approved by the Biobank Research Ethics Committee of the UMC Utrecht (Utrecht, the Netherlands) under Protocol No. 23-039 and was conducted in accordance with the Declaration of Helsinki and Dutch law. This study is compliant with all relevant ethical regulations regarding research involving human participants. Written informed consent from biobank participants was obtained.

## Author contributions

A. J. C. R.-S. and V. A. V. contributed equally to conceptualization, investigation, methodology, formal analysis, software, visualization, validation, project administration, and writing of the original draft and review and editing of the manuscript. L. R. contributed to methodology, investigation, and writing – review and editing. B. B. contributed to investigation and methodology. C. P. C. contributed resources and writing – review and editing. J. D. contributed resources. M. C. V., P. Y. W. D., and G. G. S. contributed to conceptualization, supervision, funding acquisition, and writing – review and editing. All authors reviewed and approved the final manuscript.

## Conflicts of interest

P. Y. W. Dankers is co-founder and shareholder of UPyTher and VivArt-X (two spin-off companies from the group), and CEO of SyMO-Chem.

## Supplementary Material

BM-OLF-D6BM01010E-s001

## Data Availability

The data necessary to evaluate the findings of this study are included in the manuscript and its supplementary information. The supplementary information is available at: https://doi.org/10.1039/d6bm01010e. Additional underlying data and analysis code generated during this study are available from the corresponding author upon request.
