# Supplementary material for: Integrin-guided development of defined supramolecular hydrogels for human kidney tubuloid culture
Source: Biomater Sci. 2026 Nov 3. Online ahead of print. doi: 10.1039/d6bm01010e (PMC13747057; doi:10.1039/d6bm01010e)
Supplement: Supplementary file 1 [file BM-OLF-D6BM01010E-s001.pdf]

Supplementary Information (SI) for Biomaterials Science.
This journal is © The Royal Society of Chemistry

Supporting Information

Integrin-Guided Development of Defined Supramolecular Hydrogels for Human Kidney Tubuloid Culture

Received 00th January 20xx,
Accepted 00th January 20xx

DOI: 10.1039/x0xx00000x

Aurelius J. C. Roskothen-Shevchuk,* [a] Victor A. Veenbrink,*[b,c] Laura Rijns,[b,c] Bram Bakker, [b,c] Carla Pou Casellas,[a,d] Jarno Drost, [e,f] Marianne C. Verhaar,[a] Patricia Y. W. Dankers,[b,c,g] Gisela G. Slaats **[a]

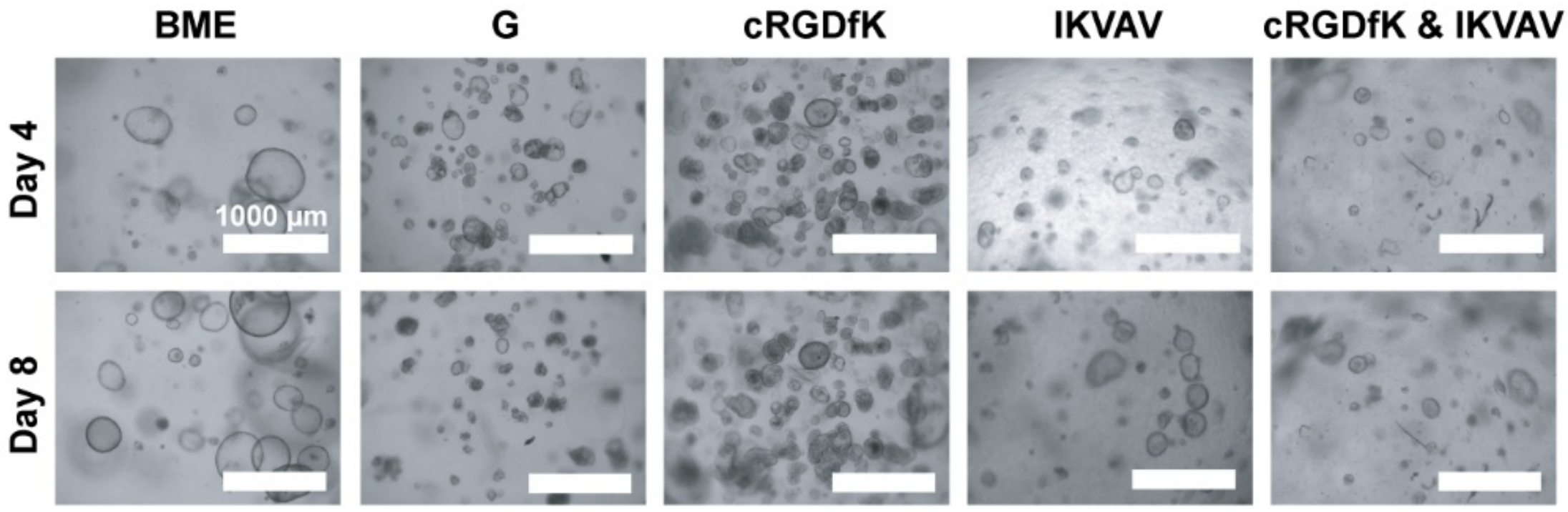

Figure S1. Brightfield images of tubuloids cultured in bulk hydrogels for 4 or 8 days. BME and non-functionalized UPy-G (G) served as the control conditions. Brightfield imaging at day 8 provides a qualitative comparison of cystic morphology across conditions, highlighting loss of cystic morphology in G but retention in BME, IKVAV, cRGDfK, and cRGDfK & IKVAV. All UPy hydrogels were formulated at an 80:1 M:B UPy molar ratio. M-type compositions were: G, 100 mol% UPy-G; IKVAV, 10.66 mol% UPy-IKVAV + 89 mol% UPy-G; cRGDfK, 11 mol% UPy-cRGDfK + 89 mol% UPy-G; and cRGDfK & IKVAV, 10.66 mol% UPy-IKVAV + 10.66 mol% UPy-cRGDfK + 78 mol% UPy-G (corresponding to 0, 1 or 2 mM UPy-peptide with 9.38, 8.38 or 7.38 mM UPy-G, respectively). Total polymer content (% w/v) was adjusted per formulation to match a UPy-G reference gel at 1.25% (w/v). Representative images are shown for donor 4 (two technical replicates). Scale bar: 1000 µm.

[a] *Department of Nephrology and Hypertension, University Medical Center Utrecht, Utrecht, The Netherlands.*
[b] *Institute for Complex Molecular Systems, Eindhoven University of Technology, Eindhoven, The Netherlands*
[c] *Department of Biomedical Engineering, Eindhoven University of Technology, Eindhoven, The Netherlands.*
[d] *Hubrecht Institute for Developmental Biology and Stem Cell Research, Royal Netherlands Academy of Arts and Sciences (KNAW) & University Medical Center Utrecht, Utrecht, The Netherlands.*
[e] *Oncode Institute, Princess Máxima Center for Pediatric Oncology, Utrecht, the Netherlands*
[f] *Division Cell Biology, Metabolism & Cancer, Department Biomolecular Health Sciences, Faculty of Veterinary Medicine, Utrecht University, Utrecht, The Netherlands*
[g] *Department of Chemical Engineering and Chemistry, Eindhoven University of Technology, Eindhoven, the Netherlands.*

* Authors contributed equally as first authors
** Corresponding author

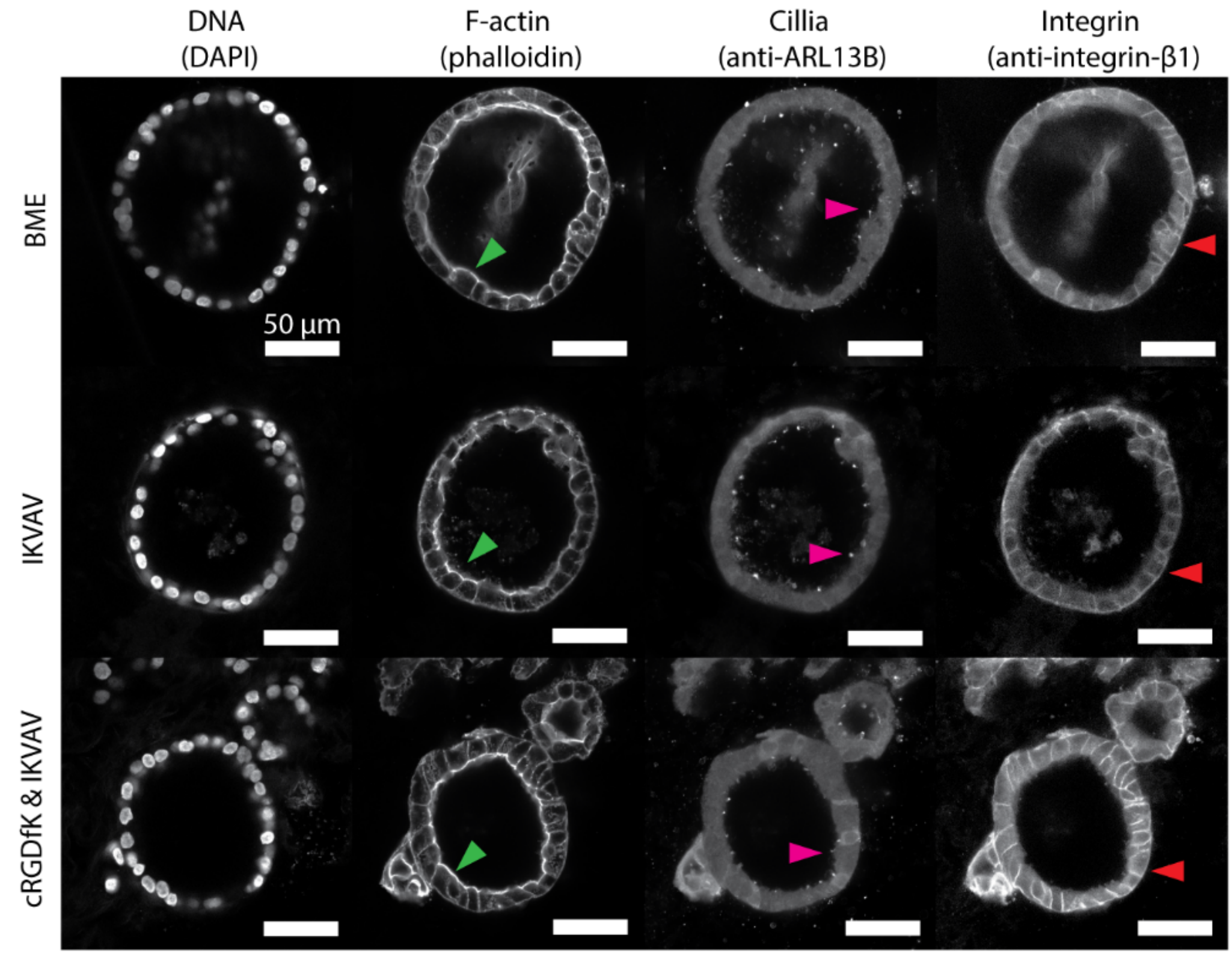

Figure S2. Apical and basal marker localization in tubuloids cultured in hydrogels. Separate fluorescence channels of Figure 3A (donor 1) show F-actin outlining the cells with apical accumulations, cilia visualized via anti-ARL13B marking the apical membrane, and integrin β1 highlighting the basal side of the tubuloids cultured in BME, or UPy hydrogels functionalized with UPy-IKVAV, or UPy-cRGDfK & UPy-IKVAV. All UPy hydrogels were formulated at an 80:1 M:B molar ratio using UPy-G with 0–2 mM UPy-peptide (IKVAV and/or cRGDfK; 10.66 mol% each when present) replacing UPy-G within the M-type fraction, and total solids were adjusted to match a 1.25% (w/v) UPy-G reference gel. Scale bar: 50 µm. Images are interpreted for marker localization only; staining intensity is not quantitative and should not be compared between conditions because hydrogel transparency and imaging depth differ between samples (see experimental section).

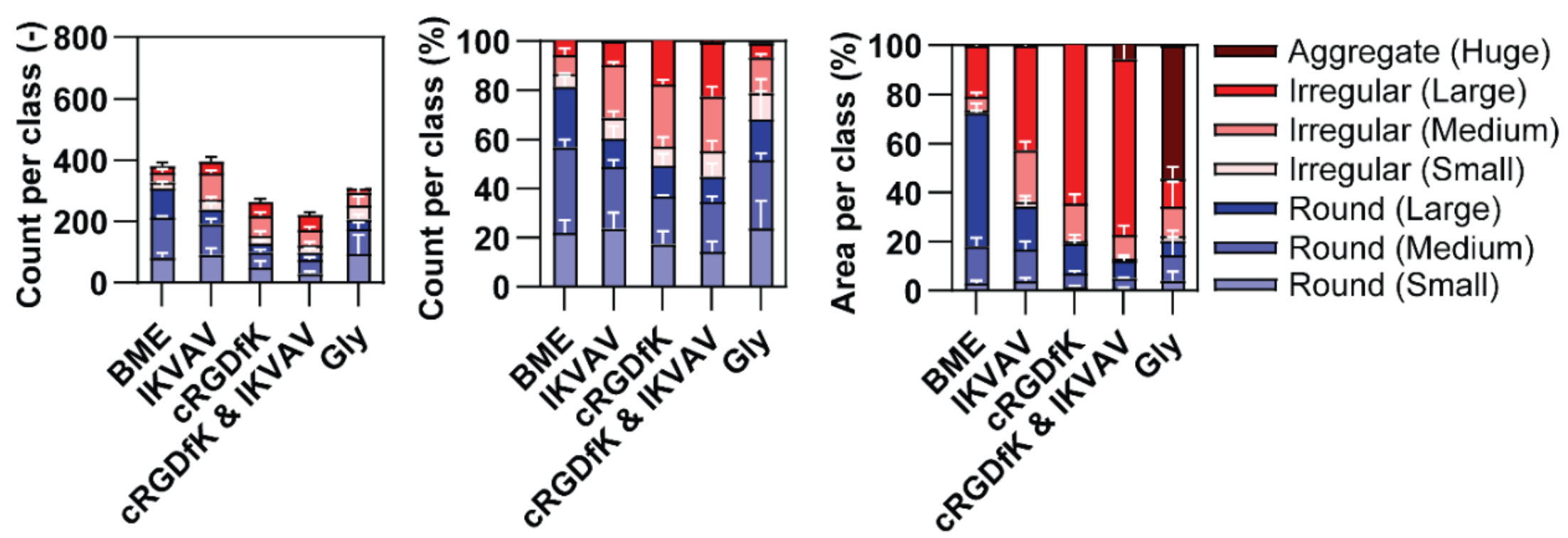

Figure S3. Morphological parameters of tubuloids under integrin β1 inhibition. Distribution of count per class and area per class for the measured outlines of all structures per image. Bars represent the mean and SD of three donors (1, 2, and 3; 179–557 outlines per donor).

 This journal is © The Royal Society of Chemistry 20xx

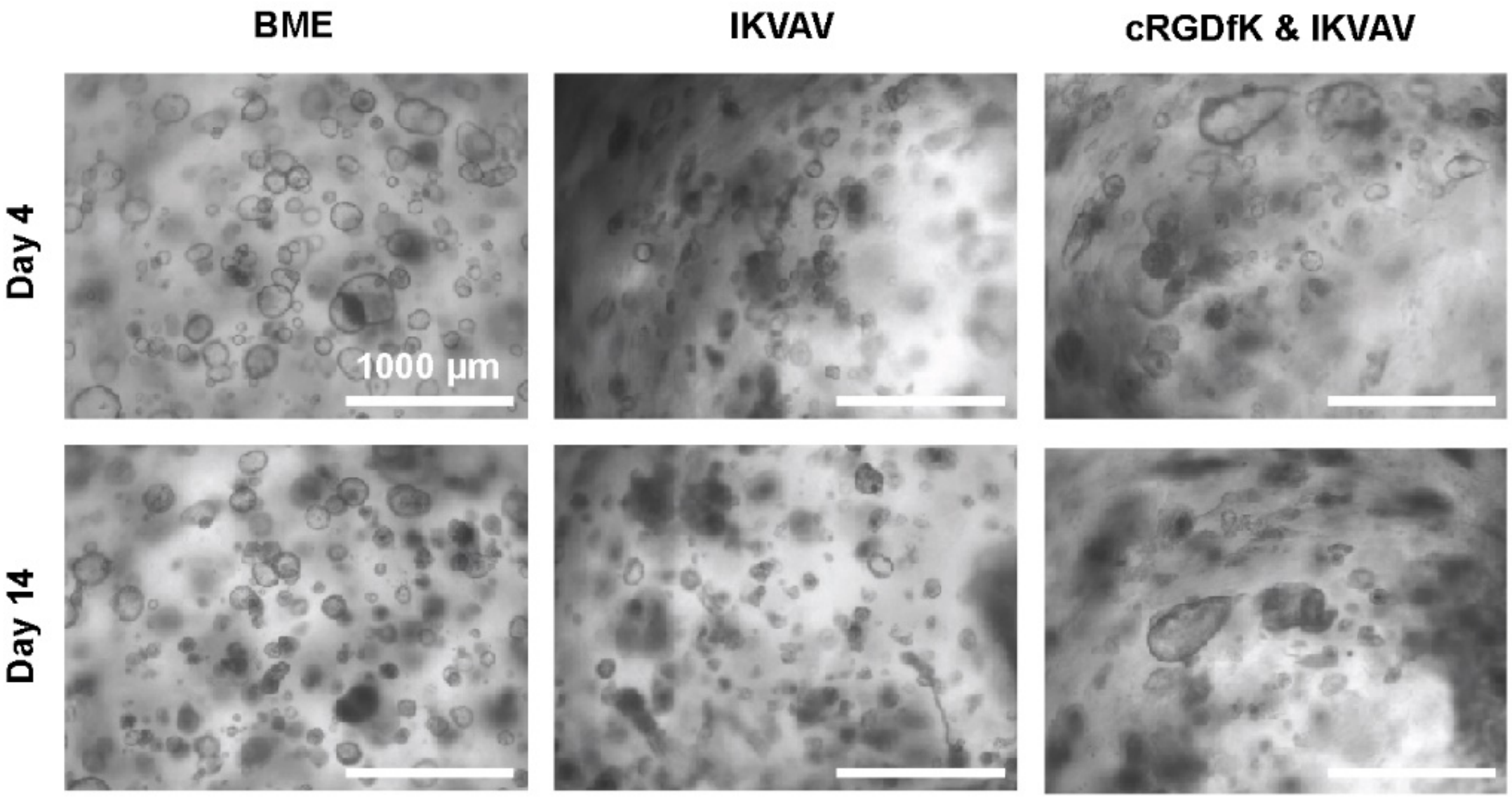

Figure S4. Brightfield images of tubuloids cultured in hydrogels over 4 or 14 days (donor 1). Tubuloids cultured in 60 µL hydrogels in U-bottom wells are shown for BME (control) and UPy hydrogels functionalized with UPy-IKVAV or UPy-cRGDfK & UPy-IKVAV. Brightfield imaging at day 14 provides a qualitative check that cystic morphology is retained over the culture period used for downstream qPCR analysis. UPy hydrogels were formulated at 80:1 M:B with UPy-G and UPy-peptide (10.66 mol% each when present); solids were matched to a 1.25% (w/v) UPy-G reference gel. Representative time-course images are shown from the same well. Scale bar: 1000 µm.

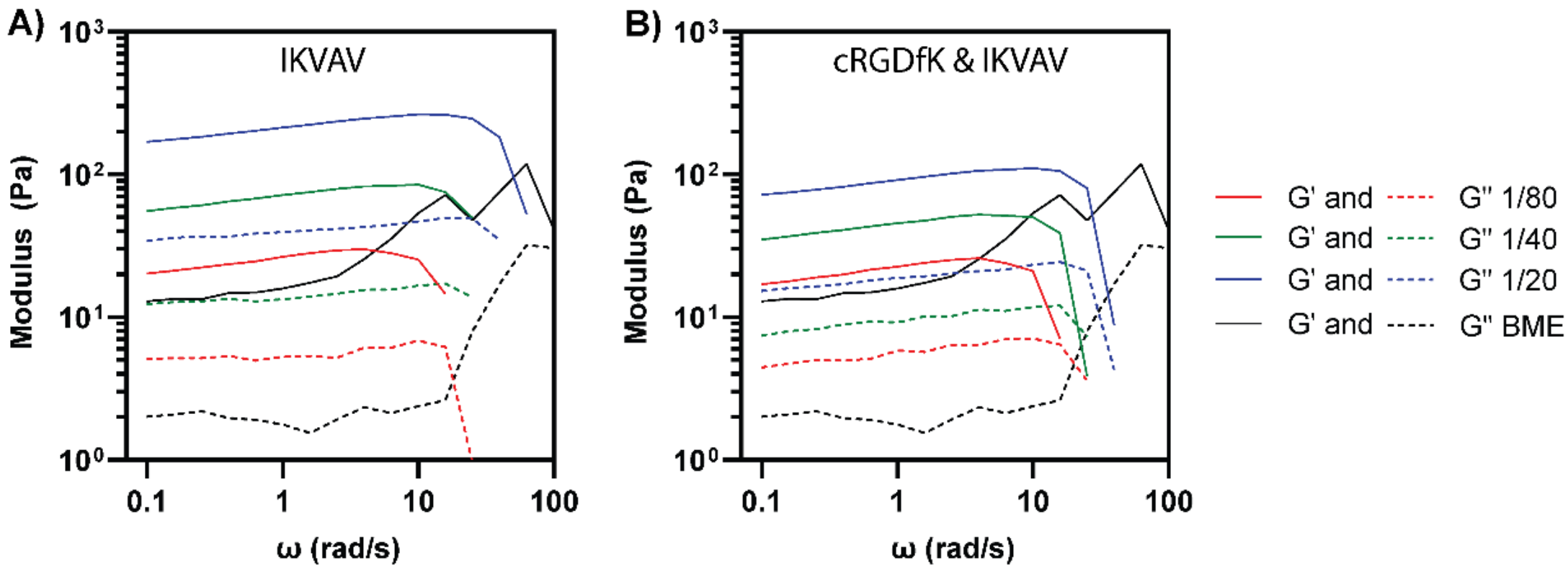

Figure S5. Frequency-dependent viscoelastic properties of the hydrogels**.** Storage modulus (G′, solid lines) and loss modulus (G″, dashed lines) of hydrogels containing (A) IKVAV or (B) cRGDfK and IKVAV at B-UPy:M-UPy ratios of 1/80 (red), 1/40 (green), and 1/20 (blue). Basement membrane extract (BME; black) is included in both panels for comparison. Frequency sweeps were performed from 100 to 0.1 rad s^{-1} at 1% strain and 37 °C. Curves represent the mean of three separately prepared gels.

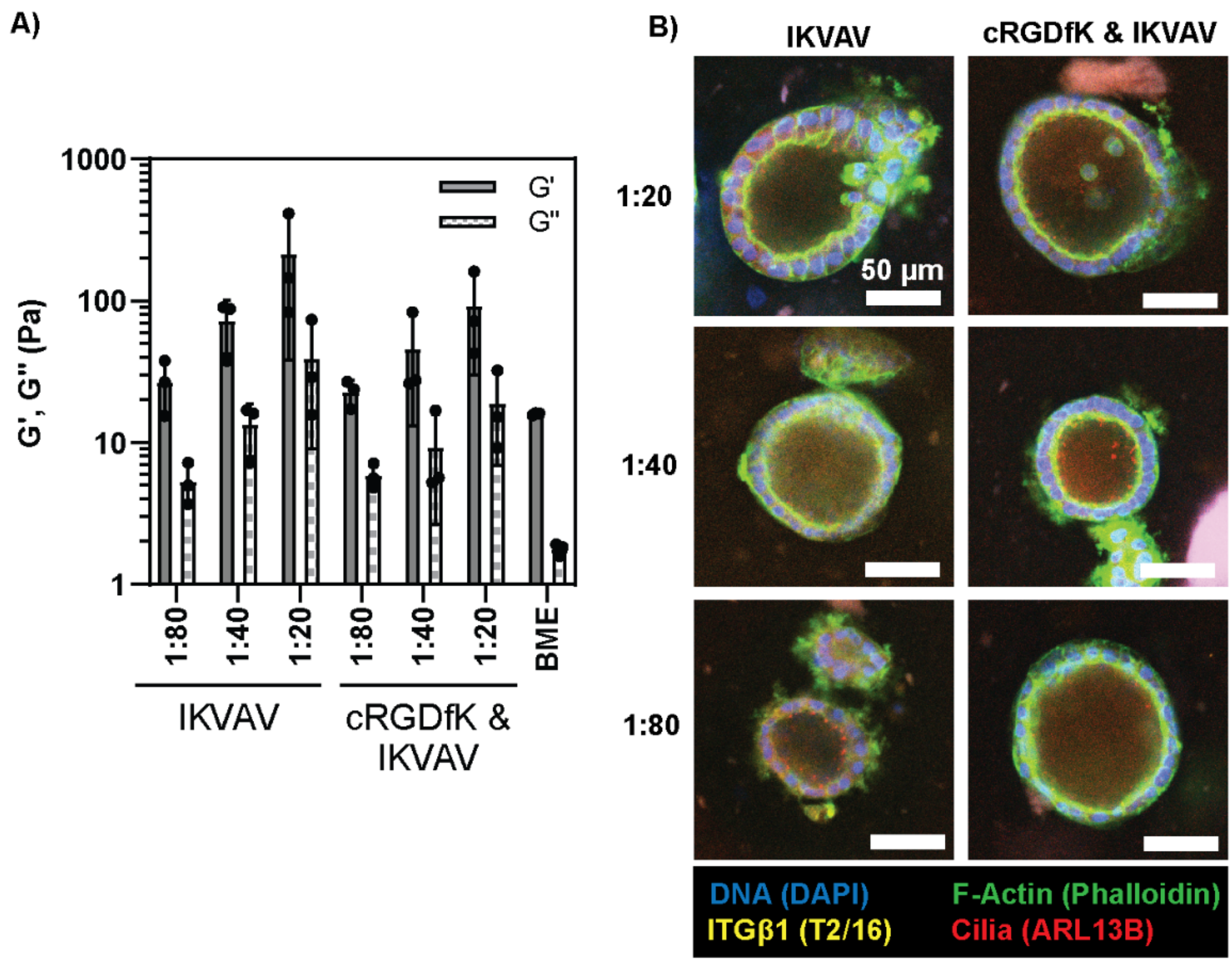

Figure S6. Tubuloids cultured in varying hydrogel stiffnesses in UPy-IKVAV and UPy-cRGDfK & UPy-IKVAV. A) G' and G" at 1 rads^{-1} and 1% strain of UPy hydrogels with an increasing amount of B-UPy and BME. Bars represent the mean and SD of three technical replicates. The data for 1:80 UPy hydrogels and BME were reproduced from figure 6B for comparison. B) Representative images of tubuloids cultured in UPy-Gels with an increasing amount of B-UPy, showing correct polarity. Samples were stained for nuclei (DAPI, blue), F-actin (phalloidin, green), integrin β1 (TS2/16, yellow), and cilia (anti-ARL13B, red). All images represent donor 5. Scale bar = 50 µm.

This journal is © The Royal Society of Chemistry 20xx
